# Enhanced Carotenoid Production in *Chlamydomonas reinhardtii* by Overexpression of Endogenousand Exogenous Beta-Carotene Ketolase (*BKT*) Genes

**DOI:** 10.3390/ijms241411382

**Published:** 2023-07-13

**Authors:** Yuanhao Chen, Hong Du, Honghao Liang, Ting Hong, Tangcheng Li

**Affiliations:** 1 Guangdong Provincial Key Laboratory of Marine Disaster Prediction and Prevention, Shantou University, Shantou 515063, China; 20yhchen5@stu.edu.cn (Y.C.);; 2 Southern Marine Science and Engineering Guangdong Laboratory, Guangzhou 510000, China; 3 Guangdong Provincial Key Laboratory of Marine Biotechnology, STU-UNIVPM Joint Algal Research Center, Institute of Marine Sciences, Shantou University, Shantou 515063, China

**Keywords:** *Chlamydomonas reinhardtii*, heterotrophy, overexpression, carotenoids, β-carotene, astaxanthin, β-carotene ketolase, *BKT*

## Abstract

*Chlamydomonas reinhardtii* is a unicellular green alga that can grow heterotrophically by using acetate as a carbon source. Carotenoids are natural pigments with biological activity and color, which have functions such as antioxidant, anti-inflammatory, vision protection, etc., and have high commercial value and prospects. We transformed *Chlamydomonas reinhardtii* with the *BKT* genes from *Phaffia rhodozyma* (*PrBKT*) and Chlamydomonas reinhardtii (*CrBKT*) via plasmid vector, and screened out the stable transformed algal strains C18 and P1. Under the condition that the cell density of growth was not affected, the total carotenoid content of C18 and P1 was 2.13-fold and 2.20-fold higher than that of the WT, respectively. *CrBKT* increased the levels of β-carotene and astaxanthin by 1.84-fold and 1.21-fold, respectively, while *PrBKT* increased them by 1.11-fold and 1.27-fold, respectively. Transcriptome and metabolome analysis of C18 and P1 showed that the overexpression of *CrBKT* only up-regulated the transcription level of *BKT* and *LCYE* (the gene of lycopene e-cyclase). However, in P1, overexpression of *PrBKT* also led to the up-regulation of *ZDS* (the gene of ζ-carotene desaturase) and *CHYB* (the gene of β-carotene hydroxylase). Metabolome results showed that the relative content of canthaxanthin, an intermediate metabolite of astaxanthin synthesis in C18 and P1, decreased. The overall results indicate that there is a structural difference between *CrBKT* and *PrBKT*, and overexpression of *PrBKT* in *Chlamydomonas reinhardtii* seems to cause more genes in carotenoid pathway metabolism to be up-regulated.

## 1. Introduction

Carotenoids are widely occurring pigments in nature, mainly found in higher plants, algae, cyanobacteria, fungi, and some non-photosynthetic bacteria [1]. Carotenoids belong to terpenoids whose structure consists of eight isoprenoids with a basic skeleton of C_40_H_56_ and are fat-soluble pigments. Carotenoids are mainly divided into carotenes and lutein, and the structural composition of carotenes consists of only two atoms, carbon and hydrogen, whereas lutein consists of three atoms: carbon, hydrogen and oxygen (oxygen functional groups). α-, β- and γ-Carotene and lycopene belong to carotenoids, whereas lutein, zeaxanthin and astaxanthin, which is a market hotspot, all belong to oxygenated carotenoids [2]. Among these carotenoids, astaxanthin is currently one of the most commercially valuable pigments of the carotenoid market due to its intense orange-red color and much higher antioxidant activity than that of other carotenoids and their derivatives. Astaxanthin is a ketocarotenoid whose structure consists of four isoprenoids with hydrophilic hydroxyl-bearing ones at both ends of the β-viologen ring; the molecular formula is C_40_H_52_O_4_. Previous studies have shown that astaxanthin has a higher antioxidant activity than β-carotenoid and α-tocopherol do [3], while astaxanthin has also been shown clinically to have anticancer, anti-Alzheimer, vision preservation, and skin aging prevention functions. Studies on Astaxanthin absorption in humans have shown that astaxanthin as a dietary supplement also provides good effects to human health via neutralizing singlet oxygen, scavenging free radicals, and inhibiting lipid peroxidation, among other effects [4].

Carotenoids mainly constitute capture antenna complexes in higher plants and play an important role in photosynthesis. Animals (including fish, crustaceans and mammals) cannot synthesize carotenoids by themselves. For example, the human eye contains lutein and zeaxanthin, constituting the macula of the human retina that functions to protect vision [5]. Humans therefore need carotenoid supplementation from food. The workhorse ‘products’ of the current carotenoid market are β-Carotene and astaxanthin (https://www.fortunebusinessinsights.com/industry-reports/carotenoids-market-100180, accessed on October 2020), which are mainly applied in the feed, health products, and cosmetic industries. The growing demand for production has created great opportunities for the market, and the continuous development of technologies for the microbial production of carotenoids in recent years has brought new opportunities to the expanding market. *Haematococcus pluvialis* and *Phaffia rhodozyma* are among the first two microorganisms to be applied to produce astaxanthin, with *Dunaliella* is mainly used for the production of β-carotenes [6,7,8]. Microalgae, as one of the most potential biological factories at present, can produce large amounts of high-value metabolites. Through the heterotrophic cultivation of microalgae, the microalgal growth environment can be controlled in a closed environment, and a large amount of biomass is harvested after continuous culture at a high concentration [9]. The heterotrophic safety, controllability, short harvest time, and high yield of microalgae are currently well-established for commercial production. The use of microalgae for the heterotrophic production of carotenoids is one of the current hotspots due to the development of microalgal heterotrophic culture techniques [10]. For example, *Haematococcus pluvialis* can obtain biomass containing 1.5–3% astaxanthin via heterotrophic cultivation in acetate [11].

*Chlamydomonas reinhardtii*, a single-cell model microalga with complete genome sequencing and various types of mutant studies, is an ideal operational object for the genetic engineering of microalgae [12]. In May 2022, the National Health Commission of the People’s Republic of China approved *Chlamydomonas reinhardtii* (heterotrophic) as a new food material, indicating that commercialized *Chlamydomonas reinhardtii* has entered a new hot flush. The protein content of *Chlamydomonas reinhardtii* powder obtained via heterotrophic culture in fermenters was greater than 30% [13]. The carotenoid synthesis pathway of *Chlamydomonas reinhardtii* is a non-mevalonate (MEP) pathway. Isoprenoids, the main structural isoprenoids, are synthesized from isopentenyl diphosphate (IPP) and its isomer dimethylallyl diphosphate (Dmapp). IPP in turn condenses with Dmapp to generate geranylgeranyl diphosphate (GGPP) as a synthetic carotenoid substrate via geranyl diphosphate (GPP) and farnesyl diphosphate (FPP). Geranylgeranyl diphosphate GGPP) is sequentially converted into lycopene by phytoene synthase (PSY), phytoene dehydrogenase (PDS) and ζ-carotene desaturase (*ZDS*). Lycopene is then synthesized separately into α-carotene (lycopene β Cyclase and lycopene ε Cyclase) and β-carotene (lycopene ε Cyclase). The main products downstream of carotene are zeaxanthin and astaxanthin. Astaxanthin in *Chlamydomonas reinhardtii* mainly passes through the β-carotenoid hydroxylases and β-carotenoid ketogenases for stepwise synthesis [14].

In *Chlamydomonas reinhardtii*, there have been numerous studies in which the directed engineering of carotenoids is performed for high-yield pigment producing algal strains. Beak et al. [15], who accumulated zeaxanthin via the method of CRISPR RNP and added it to the feed of chickens, showed that zeaxanthin in eggs also increased markedly. Tateki et al. [16] overexpressed DnaJ such as the chaperone in *Chlamydomonas reinhardtii*, and the colonies produced a 1.9-fold increase in lutein and a 1.7-fold increase over that of wild-type β-Carotene, whereas other genes in the *Chlamydomonas reinhardtii* carotenoid pathway, such as *ZDS*, lycogene epsilon cyclase (*LCYE*) and *BKT* were all featured in overexpression studies indicating increased carotenoid production from colonies. Among these, β-carotenoid ketogenases are key enzymes in the astaxanthin synthetic pathway of *Chlamydomonas reinhardtii*. It has been shown that overexpression in *Haematococcus pluvialis* can enhance astaxanthin production [17,18]. However, different β-carotenoid ketogenase genes and the cultivation mode can cause the obtained strains to differ. Therefore, we overexpressed the endogenous *Chlamydomonas reinhardtii BKT* gene and the exogenous *Phaffia rhodozyma BKT* gene, and the colonies were heterotrophic, to explore the effects of overexpression and the metabolic pathway changes of the colonies.

## 2. Results

### 2.1. Vector Construction

**Figure 1:**
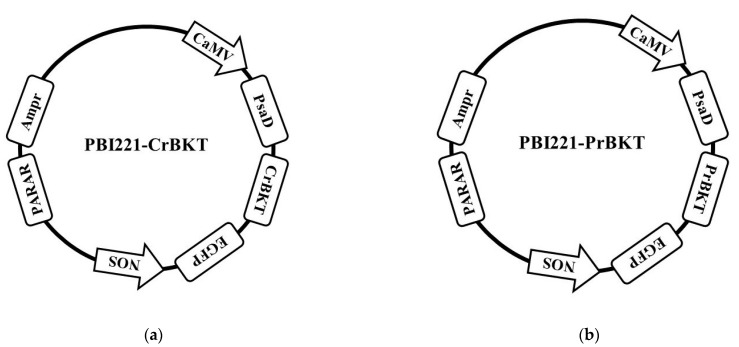
Constructed vector maps of PBI221-*CrBKT* (**a**) and PBI221-*PrBKT* (**b**). CaMV: CaMV 35S promoter, strong constitutive promoter from cauliflower mosaic virus; PsaD: *Chlamydomonas reinhardtii* photosystem I reaction center subunit II; *CrBKT*: *Chlamydomonas reinhardtii* β-Carotenoid ketogenese coding sequence after codon optimization; *PrBKT*: *Phaffia rhodozyma* β-Carotenoid ketogenese coding sequence after codon optimization; EGFP: enhanced green fluorescent protein; NOS: NOS terminator, nopaline synthase terminator and poly(A) signal; PARA: paromomycin resistance gene (including promoter); Ampr: ampicillin resistance gene (including promoter).

The designed vectors are shown as constructed maps (Figure 1), and the two vectors were named PBI221-*CrBKT* and PBI221-*PrBKT*. The paromomycin resistance gene (PARA) fragments were obtained via PCR in *E. coli* maintained in our laboratory, and the corresponding agarose gel electrophoresis is shown in Supplementary Figure S1. Gene fragments of PARA, *CrBKT* and *PrBKT* were sequenced by Nanjing Genscript Biotechnology (Nanjing, China) to ensure the same sequences as those designed. Detailed sequence information is presented in Supplementary Material S1. The sequencing results showed that none of the gene fragments had mutations, which conformed to the designed sequences.

### 2.2. Transformation of CrBKT and PrBKT

**Figure 2:**
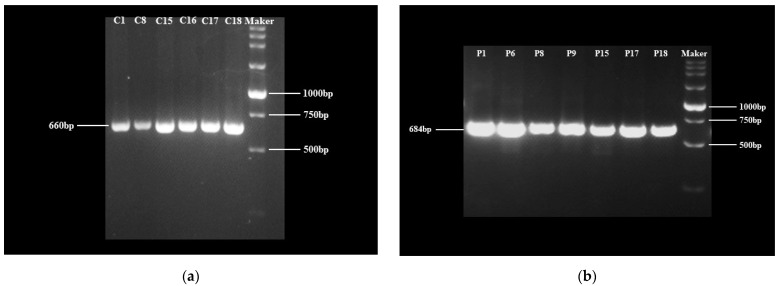
Agarose gel electrophoreses of *CrBKT* (**a**) and *PrBKT* (**b**) PCR products. (**a**) *CrBKT* PCR products signed at 660 bp. (**b**) *PrBKT* PCR products signed at 684 bp.

Via inverted fluorescence microscopy, we photographed the EGFP fluorescence signal of some colonies (Supplementary Material S2). As described in Section 4.4, finally we obtained six antibiotic-resistant colonies (group C: C1, C8, C15, C16, C17 and C18) for PBI221-*CrBKT* and 7 antibiotic-resistant colonies (group P: P1, P6, P8, P9, P15, P17 and P18) for PBI221-*PrBKT*. We performed PCR on these 13 colonies, and agarose gel electrophoresis of the PCR products showed a single band of 660 bp and 684 bp (Figure 2).

**Figure 3:**
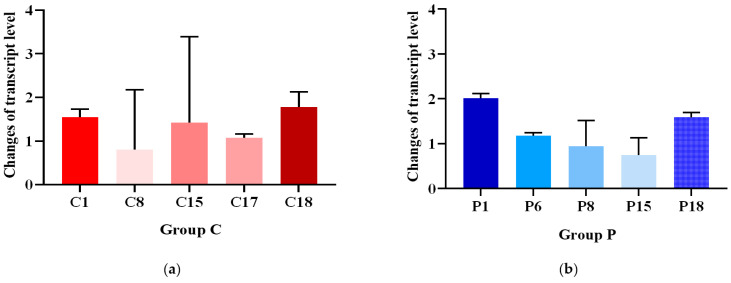
Heatmap for showing changes in gene transcript levels. (**a**) Heatmap of *CrBKT* gene transcript level changes in Group C. (**b**) Heatmap of *PrBKT* gene transcript level changes in Group P.

These 13 colonies were subcultured by plating them onto TAP (tris–acetate–phosphate medium) plates containing paromomycin (hereinafter referred to as TAP-PARA) for five passages. Three colonies, C16, P9 and P17, gradually lost their resistance to paromomycin when passaged on TAP-PARA. Then, total RNA was extracted separately for the remaining 10 colonies, and qRT-PCR of overexpressed genes (*CrBKT*, *PrBKT*) was performed after reverse transcription. The results showed that in group C, C18 had the highest *CrBKT* gene transcription level, while in group P, P1 had the highest *PrBKT* gene transcription level (Figure 3). Therefore, we selected two colonies, C18 and P1, for subsequent experiments.

### 2.3. Growth and Carotenoid Production in C. reinhardtii Colonies and Wild Type

Heterotrophic cultivation was performed for CC-125, C18 and P1. The growth cycle of these three groups was about 15 days, and the initial cell density was ~1 × 10^5^ cell/mL. The results showed that after Day 10, the growth of these three groups entered a stable phase, so a period of 0–10 days for heterotrophic cultivation was chosen to study the growth and pigment contents.

**Figure 4:**
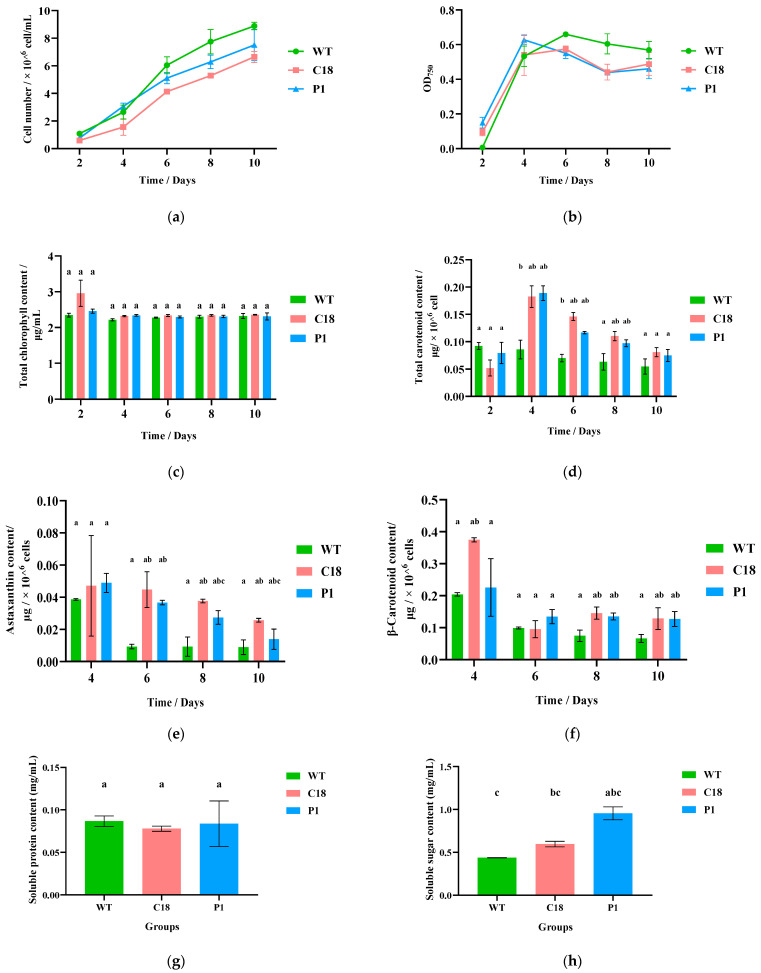
Growth curves and carotenoid production figures of CC-125, C18 and P1. (**a**) Growth curves. (**b**) Line chart for OD_750_ of the three groups. (**c**) Bar graph of total chlorophyll content. (**d**) Bar graph of total carotenoid content. (**e**) Line chart of astaxanthin content. (**f**) Line chart of β-carotenoid content. (**g**) Bar graph of soluble protein content. (**h**) Bar graph of soluble sugar content.

In the CC-125, C18 and P1 culture, the cell density increased from ~0.7 × 10^5^ cell/mL to ~80 × 10^5^ cell/mL after 10 days. The growth rate and cell density of CC-125 were slightly greater than those of C18 and P1, and reached 8.87 × 10^6^ cell/mL at Day 10. Meanwhile, the cell density of C18 and P1 reached 6.64 × 10^6^ cell/mL and 7.52 × 10^6^ cell/mL, respectively (Figure 4a). For *Chlamydomonas reinhardtii* cell growth, we also examined the OD_750_ of the cultures. As shown in Figure 4b, the OD_750_ values of the three groups stayed at a stable level from Day 4, and maintained between 0.44 and 0.66 overall.

For pigment content, we used both a UV spectrophotometer (for total chlorophyll content and total carotenoid content) and HPLC (for astaxanthin content and β-carotenoid content) for detection. The total chlorophyll content was maintained between 2 and 3 μg/mL for all three groups under heterotrophy, indicating that the cells lacked photosynthesis and that the chlorophyll content did not change with increasing days of cultivation (Figure 4c). However, there was an obvious change in the total carotenoid content, and we can see that the total carotenoid content of C18 and P1 reached a maximum (0.1825 and 0.1889 μg/10^6^ cell, respectively) at Day 4, followed by a slow decrease at Day 4–10 (Figure 4d), while the total carotenoid content of CC-125 was constantly decreasing. After Day 4, the total carotenoid content in both the C18 and P1 groups was greater than that of the CC-125 strain. Taking Day 4 as a node, the total carotenoid contents of C18 and P1 were 2.13-and 2.20-fold that of CC-125, respectively.

Qualitative and quantitative analysis of astaxanthin and β-carotenoid from cultures of CC-125, C18 and P1 was performed via HPLC. The standard curve of astaxanthin and β-carotenoid and a figure of the absorption peak for cultures are shown in Supplementary Material S3. Due to the low pigment content of partial samples at Day 2, astaxanthin and β-carotenoid were not detected. Therefore, we chose to show the data from Day 4 to 10. At Day 4, the contents of astaxanthin and β-carotenoid in all three groups were highest, and then decreased slowly. For astaxanthin (Figure 4e), C18 had the highest content, P1 was next and CC-125 had the lowest. Additionally, taking Day 4 as a node, the astaxanthin contents of C18 and P1 were 0.047 and 0.049 μg/10^6^ cells, respectively. Meanwhile, the astaxanthin content of CC125 was 0.039 μg/10^6^ cells. This indicated that the astaxanthin contents of C18 and P1 were 1.21- and 1.27-fold that of CC-125, respectively. Interestingly, at Day 6, the astaxanthin contents of C18 and P1 had a very large gap with that of CC-125, and were 5.00- and 4.11-fold of CC-125, respectively.

Similarly, C18 and P1 also had a greater β-carotenoid content than CC-125 did at Day 4–10 under heterotrophic cultivation (Figure 4f). Then, we calculated the data of Day 4; the β-carotenoid content of CC-125, C18 and P1 was 0.204, 0.375 and 0.226 μg/10^6^ cells, respectively. The β-carotenoid contents of C18 and P1 were 1.84- and 1.11-fold that of CC-125, respectively. Subsequently, the β-carotenoid content decreased greatly from Day 4 to Day 6. The β-carotenoid content after Day 6 in all three groups stabilized and the differences were not significant.

### 2.4. Transcriptome Results

#### 2.4.1. Construction and Analysis of the Transcriptome Library

As obtained via Illumina sequencing (Illumina Novaseq 6000), transcriptome sequencing data are summarized in Table 1. The WT group (CC-125), C18 group and P1 group generated approximately 42.49–51.82 million raw reads. Fastp (version 0.19.7) was used to filter the raw data, obtaining more than 39.19 million clean reads. The scores of Q30 in nine samples’ libraries were over 89.27%, indicating that all of the libraries were clean, high-quality reads. The ‘Total map’ scores were between 82.86% and 95.08%, and the ‘Unique map’ scores were between 80.43% and 92.72%. The WT group showed an overall stronger alignment than C18 and H1 did.

**Figure 5:**
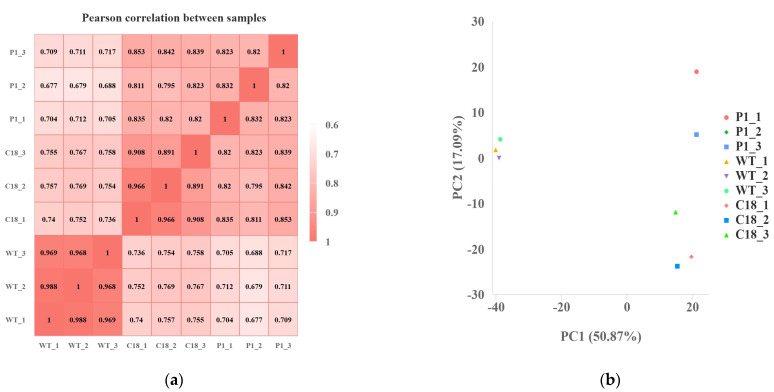
Analysis of gene expression levels. (**a**) Pearson correlation between samples of WT, C18 and P1. (**b**) PCA (principal component analysis) for samples of WT, C18 and P1.

There was a good correlation of gene expression (R^2^ > 0.968 for the WT, R^2^ > 0.891 for C18 and R^2^ > 0. 82 for P1) among the biological replicates of the three groups, indicating the reliability of the biological replicates (Figure 5a). As obtained via performing PCA (principal component analysis) on the gene expression values (FPKM) of all samples, Figure 5b shows that samples are more dispersed between groups and that samples within groups cluster as closely as possible. As can be seen from the PCA plot, the WT group is more distant from C18 and P1, while C18 was relatively close to P1, indicating that the gene expression levels of these two groups were similar to some extent.

#### 2.4.2. Differential Expression of Genes of C18 and P1 Colonies

**Figure 6:**
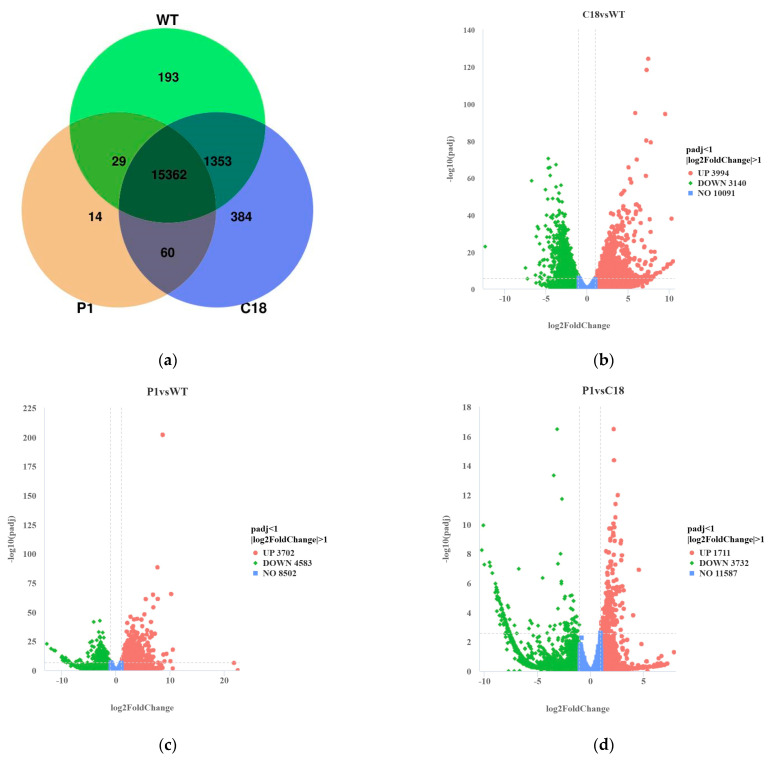
Analysis of differentially expressed genes (DEGs). (**a**) Venn diagram. (**b**) Volcano plot for DEGs of C18 vs. WT. (**c**) Volcano plot for DEGs of P1 vs. WT. (**d**) Volcano plot for DEGs of P1 vs. C18.

The differential expression of genes (DEGs) between samples was identified via|log^2^ (foldchange)| ≥ 1 & P adj ≤ 0.05. The Venn diagram (Figure 6a) exhibited that there are 620 independent DEGs in the WT. Additionally, there are 443 and 281 independent DEGs in C18 and P1, respectively. These three groups shared 11,034 DEGs. C18 and P1 shared 782 DEGs, which is more than those shared by C18 and WT (584 DEGs), and P1 and the WT (289 DEGs).

Briefly, 3994 up-regulated and 3140 down-regulated genes were identified in C18 vs. WT (Figure 6b). Likewise, transcriptome data indicated that 3702 up-regulated and 4583 down-regulated genes were identified in P1 vs. WT (Figure 6c). This revealed significant differences between DEGs from C18 versus those from P1 relative to those from the WT, while P1 vs. C18 also had 1711 up-regulated genes vs. 3732 down-regulated genes, indicating that there are also a large number of differences in gene expression levels between these two *BKT* gene-overexpressing strains.

#### 2.4.3. Analysis of Differentially Expressed Genes

**Figure 7:**
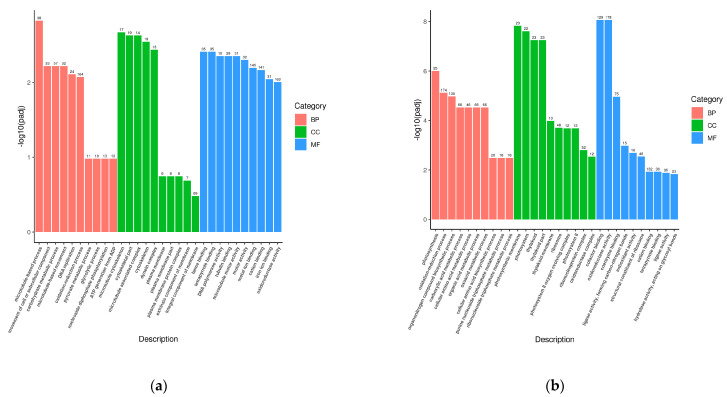

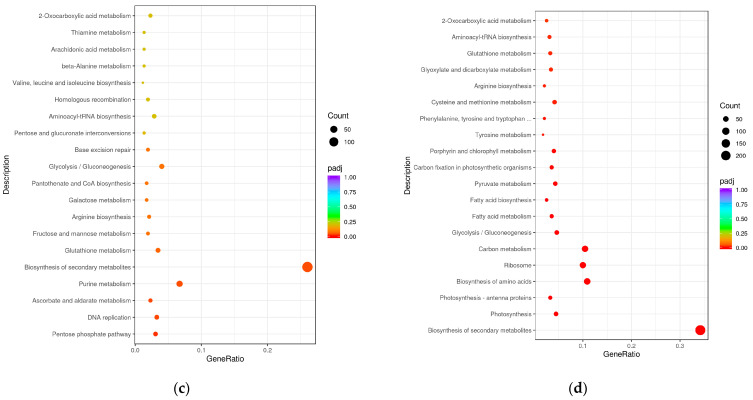
DEGs enrichment results via GO and KEGG. (**a**) GO functional classification for DEGs of C18 vs. WT. (**b**) GO functional classification for DEGs of P1 vs. WT. (**c**) KEGG pathway annotation of DEGs for DEGs of C18 vs. WT. (**d**) KEGG pathway annotation of DEGs for DEGs of P1 vs. WT.

When analyzing the DEGs among these groups, we mainly focused on C18 vs. the WT and P1 vs. the WT. First, a GO analysis (Figure 7a,b) of C18 vs. the WT revealed that DEGs were mainly enriched in the following pathways: the microtubule-based process, movement of cellular or subcellular component, carbohydrate metabolic process, etc. Under the CC (cellular component) category, these differentially expressed genes were significantly numerous in the microtubule cytoskeleton, cytoskeletal part and microtubule-associated complex. Under the MF (molecular function) category, most DEGs were enriched in heme binding, tetrapyrrole binding and DNA polymerase activity. However, P1 vs. WT was quite different, and the DEGs were mainly enriched in photosynthesis, the oxidation–reduction process, and organonitrogen compound biosynthetic process under the BP (biological process) category, while DEGs were mainly enriched in the photosynthetic membrane, photosystem and thylakoid under the CC category. Under the MF category, we can clearly see the DEGs were enriched in cofactor binding, oxidoreductase activity and coenzyme binding.

While through the KEGG-enriched bubble plot, we found more information about DEGs (Figure 7c,d). First, the KEGG enrichment data of C18 vs. WT showed that the DEGs were mainly associated with the following pathways: the pentose phosphate pathway, DNA replication, ascorbate and aldarate metabolism, purine metabolism, the biosynthesis of secondary metabolites, etc. However, DEGs of P1 vs. WT were mainly enriched in the biosynthesis of secondary metabolites, photosynthesis, photosynthesis-antenna proteins, biosynthesis of amino acids and ribosome, etc. Combining the KEGG enrichment results of these two groups, it became obvious that photosynthesis and secondary metabolite synthesis were the pathways commonly enriched by the DEGs of C18 and P1 vs. WT. Analysis of the DEGs revealed significant gene expression level differences between the C18 and P1 strains relative to that of the WT, presumably due to carotenoid metabolic pathway changes facilitating differences in other growth-related gene expression.

#### 2.4.4. qRT-PCR Validation of Key Enzyme Results

**Figure 8:**
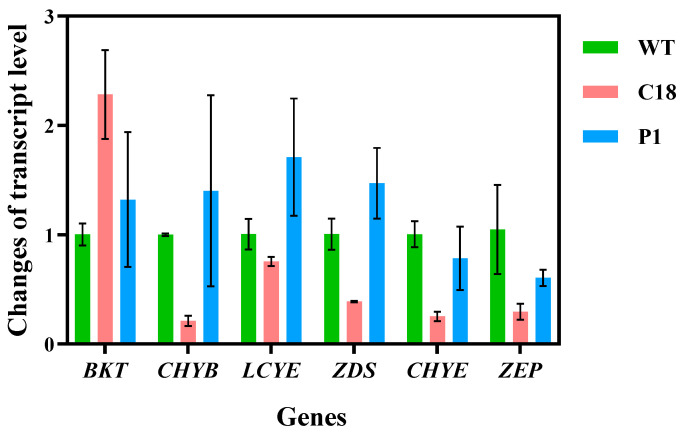
Heatmap of transcript levels. *BKT*, β-carotene ketolase; *CHYB*, β-carotene hydroxylase; *LCYE*, lycopene e-cyclase; *ZDS*, ζ-carotene desaturase; *CHYE*, carotene e-hydroxylase; *ZEP*, zeaxanthin epoxidase.

We analyzed the data from qRT-PCR, using β-actin as the reference gene. We calculated the relative expression of the target gene using the double delta Ct (ΔΔ Ct) method, and the results are shown in Figure 8. The results showed that the qRT-PCR results were consistent with the transcriptome data. We used the gene expression level of the WT to calculate the relative expression level, so that several genes in the WT had relative expression levels close to one, indicating the good reliability of the results. Except for *BKT*, all genes in the C18 group had slightly down-regulated expression levels, but not more than 0.5-fold, and there was no significant difference. While there was no significant difference in the relative expression level of *BKT* gene in P1 group, *CHYB*, *LCYE* and *ZDS* had up-regulated relative expression levels. Additionally, the results of C18 and P1 showed that *CHYE* and *ZEP* genes had down-regulated expression levels. Further analysis will be provided in the discussion.

### 2.5. Metabolome Results

#### 2.5.1. Qualitative versus Relative Quantification of Metabolites

**Figure 9:**
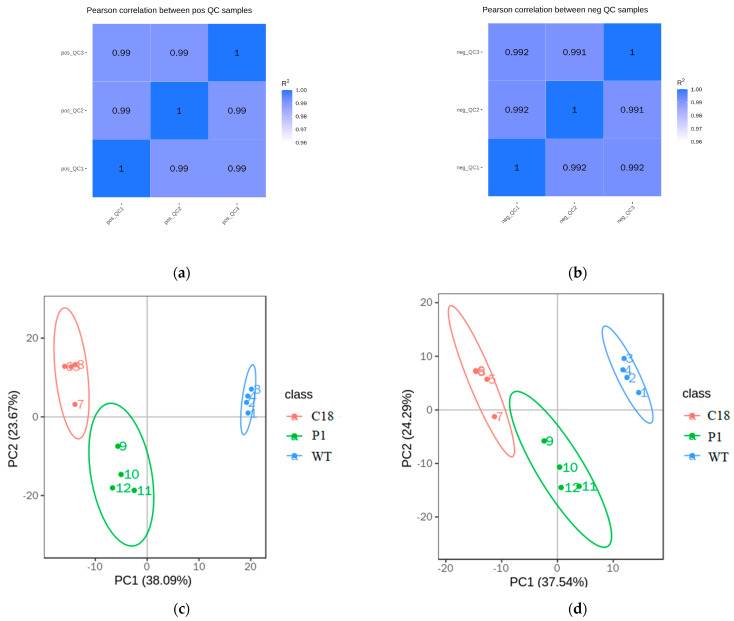
Quality control of untargeted metabolomics. (**a**) QC sample correlation analysis (positive ions). (**b**) Correlation analysis of QC samples (negative ions). (**c**) PCA analysis of total samples (positive ions). (**d**) PCA analysis of total samples (negative ions).

The Pearson correlation coefficients (R^2^) between QC samples were calculated based on the relative quantification values of metabolites, and the Pearson correlation coefficient for both positive and negative ions was ≥0.99 (Figure 9a,b). The QC samples’ R^2^ value was close to one, indicating that the metabolome detection of the samples was stable and that high-quality data were obtained. The peaks extracted from all experimental samples and QC samples were subjected to PCA analysis. The higher the difference in QC samples, the better the stability of the whole method and the higher the data quality. It is reflected in the PCA analysis chart that the distribution of QC samples will be clustered. Figure 9c,d shows that samples are more highly dispersed between groups and that samples within groups cluster as closely as possible, while C18 is relatively close to P1, indicating that the detected metabolites of these two groups are similar to a certain extent.

#### 2.5.2. Main Differential Metabolite Comparisons

Differential metabolites were screened according to three parameters of VIP (variable importance in the projection), FC (fold change) and *p*-value (by *t*-test), setting VIP > 1.0, FC > 1.5 or FC < 0.667 and *p*-value < 0.05; the differential metabolites screened are shown in Table 2. It is interesting that the total metabolite identification results were the same number in two comparison groups, C18 vs. WT and P1 vs. WT. However, the total number of significantly different metabolites, including the number of up- and down-regulated metabolites, was somewhat different. The number of significantly differential metabolites of C18 vs. WT was slightly more than that for P1 vs. WT (240 > 222, 136 > 113). Additionally, the number of metabolites with a significant difference was more for positive ions than for negative ions in both comparison groups (240 > 136, and 222 > 113). The similar number rules of the two groups indicated that the differential metabolites of C18 versus P1 for the two groups relative to the WT were similar. This represents a degree of similarity between the two groups of C18 and P1 at the metabolic level.

#### 2.5.3. Pathway Enrichment of Differential Metabolites

**Figure 10:**
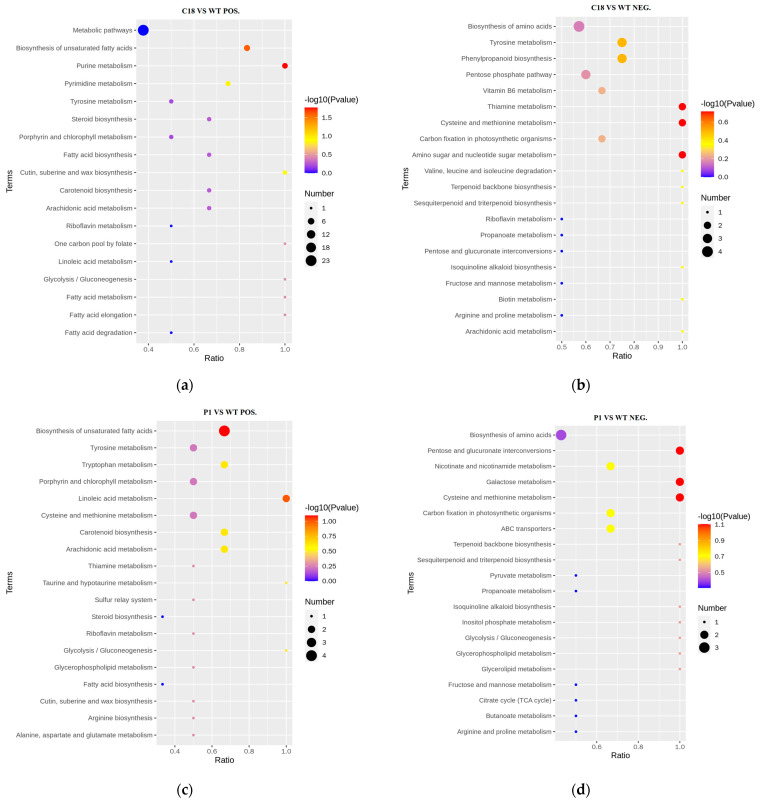
KEGG enrichment results of differential metabolites. (**a**) Scatter plot of KEGG enrichment results for differential positive ion metabolites in C18 vs. WT. (**b**) Scatter plot of KEGG enrichment results for differential negative ion metabolites in C18 vs. WT. (**c**) Scatter plot of KEGG enrichment results for differential positive ion metabolites in P1 vs. WT. (**d**) Scatter plot of KEGG enrichment results for differential negative ion metabolites in P1 vs. WT.

From the KEGG enrichment results of differential metabolites, the number of differential metabolite enrichments on each pathway for C18 and P1 vs. WT was not very large (Figure 10). Although 23 differential metabolites (pos) in C18 vs. WT were enriched in metabolic pathways, the corresponding *p*-value was close to 0, which is not a representative conclusion (Figure 10a). Similarly, four differential metabolites were enriched in the biosynthesis of amino acids, but the *p*-value was close to 0.2 (Figure 10b). Moreover, by checking P1 vs. WT, only a maximum of three to four differential metabolites were enriched per pathway (Figure 10c,d).

Subsequently, we focused on which pathways the differential metabolites with a high *p*-value were mainly enriched. The results indicated that the differential metabolites were mainly enriched in the biosynthesis of unsaturated fatty acids, biosynthesis and metabolism of amino acids, biosynthesis and metabolism of soluble sugars, and photosynthetic pigments. The differential metabolites of lipids, amino acids of C18 vs. WT were mainly unsaturated fatty acids, steroids, amino acid cysteine, methionine, tyrosine, etc. The pathways involved are glycolysis and amino sugar and nucleotide sugar metabolism. Additionally, the differential metabolites involved in the carotenoid pathway are terpenoids, tocopherol and canthaxanthin.

The differential metabolites in P1 vs. WT also had predominantly unsaturated fatty acids, similarly to C18 vs. WT. However, the differential amino acids were mainly enriched in the metabolism of tryptophan, cysteine, methionine, arginine and other amino acids. Regarding sugar metabolism, the differential metabolites of P1 vs. WT were additionally enriched in pentose and glucuronate interconversions and galactose metabolism. In carotenoid-related pathways, differential metabolites are enriched not only in terpenoid synthesis but also in the synthesis of sesquiterpenoid and triterpenoid. Tocopherol metabolic pathways lacked in P1 vs. WT KEGG results. Meanwhile, C18 and P1 vs. WT all had differential metabolites enriched in the porphyrin and chlorophyll metabolism.

## 3. Discussion

### 3.1. Association and Differences between Exogenous and Exogenous BKT Gene Overexpression

Overexpression in genetic engineering is a common approach to breeding improvement. In microalgae, the modification of a given gene’s genome is achieved by changing its biological properties by increasing the amount of expression of that gene [19]. Overexpression enables the increased production of specific proteins, which in turn influences the characteristics of the microalgal strain. For instance, it can lead to a higher yield of specific metabolites, ultimately enhancing the nutritional value of microalgae. Therefore, overexpression technology serves as an effective approach for improving and selecting commercially viable microalgal strains. *Chlamydomonas reinhardtii* is considered a safe and edible microalga species with a higher protein content compared to that of *Chlorella vulgaris*. Additionally, it possesses an intact genome, making it a suitable candidate for genetic manipulation [20]. Within the carotenoid metabolism pathway of *Chlamydomonas reinhardtii*, β-cartene ketolases play a crucial role. These enzymes are responsible for converting β-carotenoids through ketification reactions, leading to the formation of valuable products such as echinenone, canthaxanthin and astaxanthin, among others.

**Figure 11:**
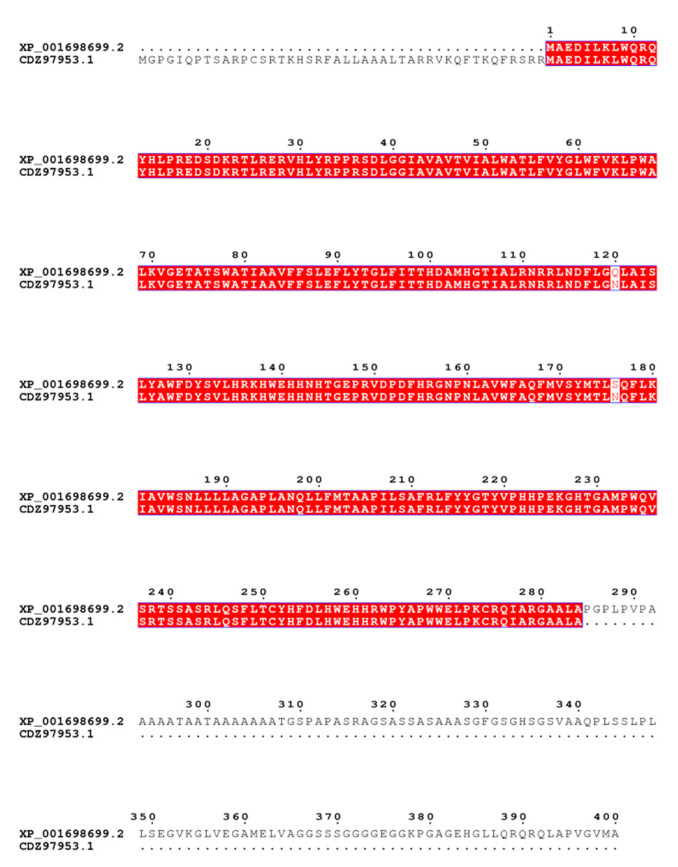
Alignment of amino acid sequences of β-carotenoid ketogeneses from *Chlamydomonas reinhardtii* (XP_001698699.2) and *Phaffia rhodozyma* (CDZ97953.1).

However, recent studies have focused on engineering the *CrBKT* gene or co overexpressing multiple genes of the carotenoid synthesis pathway to achieve high carotenoid and astaxanthin production. This study mainly focused on the *BKT* gene. We found that the *BKT* gene of *Phaffia rhodozyma* had the highest homology with *CrBKT* via alignment in NCBI. We therefore decided to overexpress *CrBKT* versus *PrBKT* separately to investigate whether or not the overexpression results were differential. From the amino acid alignment results of these two proteins (Figure 11), we can see that *PrBKT* has a longer stretch of sequences than *CrBKT* does at the beginning, and *CrBKT* has a longer stretch of sequences than *PrBKT* does at the C’ end. In the study by Federico et al., they found that there was a long, seemingly nonsense repeat in the β-carotenoid ketolase gene of *Chlamydomonas reinhardtii*. The expression of *CrBKT* will be optimized after these redundant sequences are removed, promoting astaxanthin synthesis. However, *PrBKT* has an extended sequence in the N-segment, which we consider relevant for carotenoid synthesis in yeast, and does not belong to a ‘pseudogene’. So, when initially designing the experiments, we speculated that the overexpression effect of *PrBKT* would be better than that of *CrBKT*.

Indeed, the carotenoid production level of C18 was comparable to that P1, even after codon optimization. We speculate that the copy number and insertion site of the exogenous *BKT* gene may be different from that of the endogenous gene. It contains copies of a foreign gene within *Chlamydomonas reinhardtii* or a different chromosomal location that affects its expression level and stability. Second, the translated protein of *PrBKT* may be subject to splicing and modification effects different from those of endogenous genes, such as methylation, acetylation, phosphorylation, etc., which can affect its post-transcriptional levels and function. Combined with the physiological data, we speculate that the high similarity in the amino acid sequences of these two proteins makes the expression of *CrBKT* and *PrBKT* have similar effects. However, by analyzing the transcriptome and metabolome, the two strains C18 vs. P1 showed partially different expression levels with metabolites on other metabolic pathways, which we further discuss in the following two sections.

### 3.2. Transcriptome and Metabolome Analysis and Discussion of the Carotenoid Metabolic Pathways in the C18 and P1 Strain

The function of β-carotene ketolase is mainly to ketolate the ring end groups of β-carotene. Some studies have shown that the upstream steps of carotenoid synthesis are key steps for β-carotene and astaxanthin biosynthesis [21]. However, by modifying the *BKT* gene, astaxanthin production can also be greatly increased. Functionally, if a large amount of astaxanthin needs to be synthesized in *Chlamydomonas reinhardtii*, not only does the expression level of β-carotene ketolase need to be increased, but so does the coordination of β-carotene carboxylase [22,23]. Astaxanthin synthesis in *Chlamydomonas reinhardtii* belongs to the bicyclic pathway, but this green microalga itself hardly synthesizes astaxanthin, because its β-carotene ketolase (*CrBKT*) gene expression level is very low, and it contains an uncommon C-terminal extension, which may be a pseudogene [17]. The genes for synthesizing β-carotene in *Phaffia rhodozyma* are crtYB, crtI, crtO, crtS and so on. Among them, crtI catalyzes carotenoid conversion into β-carotene. Subsequently, β-carotene can be converted into astaxanthin by *BKT* and *CHYB*. It can also be converted into astaxanthin by CrtR and CrtO. At the same time, CrtS, the protein translated after astaxanthin synthetase), can continuously catalyze the 4-ketolation and 3-hydroxylation of β-carotene, to synthesize astaxanthin [24]. However, according to Li et al.’s study, in three different *P.rhodozyma* strains, the CrtS gene was poorly correlated with astaxanthin synthesis [8,25]. However, their relationship and regulatory mechanism are not clear.

The β-carotene ketolase protein (CDZ97953.1) of *Phaffia rhodozyma* consists of 328 amino acids, with a molecular weight of 36.8 kDa and an isoelectric point of 5.18. There are 250 residues, of which 116 are polar residues and 134 are non-polar residues. Similarly, the β-carotene ketolase (β-carotene ketolase) (X_001698699.2) of *Chlamydomonas reinhardtii* consists of 400 amino acids, with a molecular weight of 39.3 kDa and an isoelectric point of 5.03, of which 108 are polar residues and 259 are non-polar residues. Therefore, whether it is *Phaffia rhodozyma* or *Chlamydomonas reinhardtii*’s *BKT*, they are relatively conservative and function-specific.

**Figure 12:**
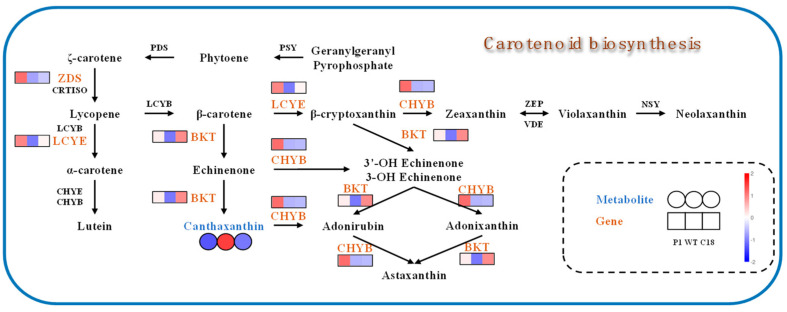
Carotenoid metabolic pathways of *Chlamydomonas reinhardtii*. *BKT*, β-carotene ketolase; *CHYB*, β-carotene hydroxylase; *CHYE*, carotene e-hydroxylase; CRTISO, carotenoid isomerase; LCYB, lycopene b-cyclase; *LCYE*, lycopene e-cyclase; NSY, neoxanthin synthase; PDS, phytoene desaturase; VDE, violaxanthin de-epoxidase; *ZDS*, f-carotene desaturase; *ZEP*, zeaxanthin epoxidase. Gene expression levels of *BKT*, *LCYE*, *ZDS* and *CHYB* in P1, the WT and C18 are presented in a heat map (square pattern). The differential metabolite canthaxanthin is shown in a heatmap (circular pattern), too.

As shown in Figure 12, we present the carotenoid pathway of C18 and P1, and indicate the transcriptome and metabolome data. In C18, the overexpression of *CrBKT* only up-regulated the transcription level of *BKT* and *LCYE*. However, in P1, the overexpression of *PrBKT* led to the up-regulation of *BKT*, *LCYE*, *ZDS* and *CHYB*. The β-carotene synthesis pathway in *Chlamydomonas reinhardtii* mainly consists of two branches; one is the β-cyclization branch catalyzed by lycopene cyclase (*LCYE*), which can convert β-carotene into β-cryptoxanthin, and then into astaxanthin, and the other is the ε-cyclization branch catalyzed by β-carotene hydroxylase (BCH), which can convert β-carotene into echinenone, and then that into canthaxanthin. The *BKT* gene belongs to monocyclic pathway, and β-carotene is synthesized into astaxanthin via the ketolation and carboxylation of *BKT* and *CHYB*. We overexpressed the *BKT* gene to enhance monocyclic pathway. The carotenoid synthesis of zeaxanthin is a bicyclic pathway. How these two pathways regulate each other is not clear. In *Chlamydomonas reinhardtii*, *LCYE* and *CHYB* have low expression levels and activity, resulting in most carotenoids existing as β-carotene in cells. In C18, we speculate that the up-regulation of *LCYE* makes more β-carotene flow to the bicyclic pathway, resulting in a decrease in the monocyclic pathway intermediate canthaxanthin. Additionally, P1, in addition to *BKT* and *LCYE* gene up-regulation, also induced *ZDS* and *CHYB* up-regulation. This was also confirmed via the qRT-PCR results. We speculate that the overexpression of the *PrBKT* gene leads to the increased synthesis of astaxanthin in cells, which affects the balance and feedback regulation of the carotenoid synthesis pathway, making the upstream components need more β-carotene; the *ZDS* gene was positively induced. Meanwhile, because Phaffia rhodozyma’s β-carotene ketolase does not contain ‘pseudo-genes’, its activity is stronger than *CrBKT*’s, so *CHYB* in the monocyclic pathway was also affected, and its gene transcription level was up-regulated. Additionally, we speculate that the up-regulation of *CHYB*, *BKT* and *LCYE* leads to β-carotene subsequently synthesizing astaxanthin as a substrate, resulting in a decrease in intermediate metabolite canthaxanthin. Notably, the results of qRT-PCR indicated that two genes, *CHYE* and *ZEP*, were down-regulated in C18 and P1. We speculate that because the substrate lycopene transports more β-Carotenoids, β-Carotenoids were allocated more to astaxanthin synthesis, which led to this result.

### 3.3. Other Metabolic Pathway Changes in C18 and P1 Strain

**Figure 13:**
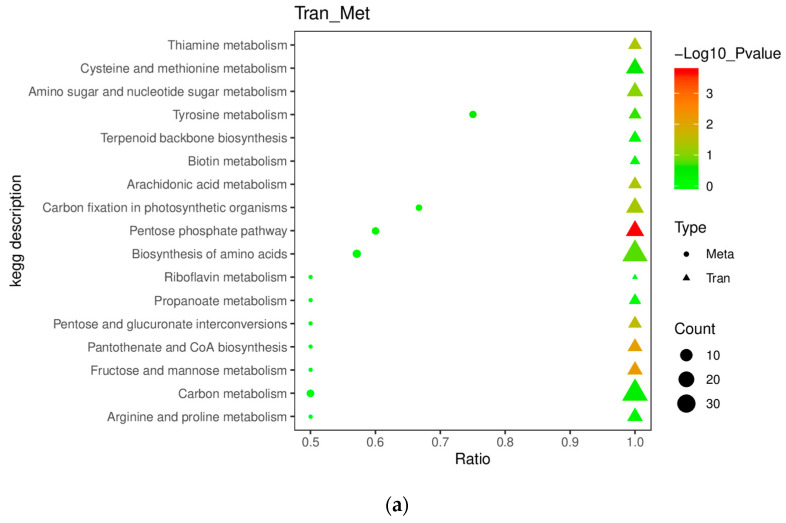

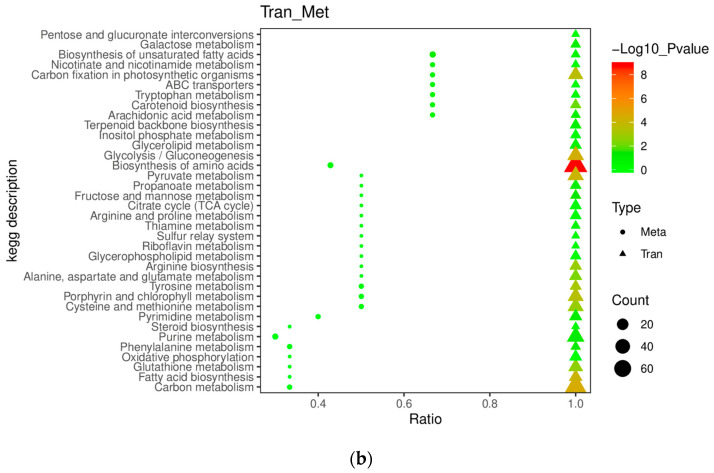
Pathway enrichment analysis for metabolic and transcriptional associations. (**a**) C18 vs. WT. (**b**) P1 vs. WT.

We correlated the transcriptome with the data of metabolome to analyze the results of the enriched pathways, and the results are shown in Figure 13.

#### 3.3.1. Porphyrin and Chlorophyll Metabolism

In *Chlamydomonas reinhardtii*, carotenoids and chlorophylls have quite a lot of connections. Carotenoids and chlorophylls are both synthesized from isopentenyl diphosphate (IPP) as a precursor, sharing part of the metabolic pathway [26,27]. The increase in carotenoids promotes the expression of chlorophyll synthesis genes. Some studies have shown that in some plants, the content of carotenoids and the expression levels of chlorophyll synthesis-related genes are positively correlated [28,29,30]. For example, in tomato, varieties with high carotenoid content also have higher expression levels of chlorophyll synthesis-related genes (such as PORA1/2, CAO, etc.) [31]. This suggests that there may be some regulatory relationship between carotenoids and chlorophylls. For example, the final product of the terpenoid skeleton is GGPP. GGPP is an important isoprenoid compound that participates in various biosynthetic pathways such as those of carotenoids, chlorophylls, steroids, etc. Yang et al. cloned and characterized the GGPP synthase (GGPS) gene responsible for carotenoid synthesis in *Pyropia umbilicalis* and found that it had different expression patterns under different light intensities [28,32]. In the process of chlorophyll synthesis, GGPP binds with chloride to form the ring structure of chlorophyll. Therefore, GGPP is one of the important components of the chlorophyll synthesis pathway.

Carotenoids and chlorophylls are both photosynthetic pigments that participate in light capture and conversion. Carotenoids can protect chlorophylls from damage caused by excess light energy or low temperature stress, playing a role in antioxidant and non-photochemical quenching. Carotenoids have different compositions and contents in *Chlamydomonas reinhardtii* at different developmental stages or growth conditions, while chlorophylls are relatively stable [20]. This is consistent with our pigment measurement data which showed that there was no significant difference in the content of chlorophylls among the WT, C18 and P1 groups at 2–10 days. However, transcriptomic and metabolomic correlation enrichment results showed that most genes involved in the porphyrin and chlorophyll biosynthesis pathway were up-regulated in C18 and P1, especially in P1.

#### 3.3.2. Terpenoid Backbone Biosynthesis

The content of carotenoids also affects the synthesis of terpenoid skeletons. The transcriptomics results showed that 8–10 genes involved in the terpenoid biosynthesis pathway were up-regulated in C18 and P1. In *Chlamydomonas reinhardtii*, carotenoids are composed of C40 tetraterpenoid skeletons, which are condensation products of isopentenyl diphosphate (IPP) and dimethylallyl diphosphate (DMAPP). IPP and DMAPP are universal precursors of terpenoid compounds, which are produced by the methylerythritol phosphate (MEP) pathway in *Chlamydomonas reinhardtii* [33]. The MEP pathway mainly occurs in chloroplasts and provides IPP and DMAPP for the synthesis of compounds such as carotenoids and chlorophylls [30]. Therefore, when the content of carotenoids increases, more IPP and DMAPP will be consumed, thus reducing the synthesis of other terpenoid compounds, and vice versa. This phenomenon is called “metabolic competition”. Consequently, the high carotenoid production detected in C18 and P1 may trigger a general increase in the expression levels of genes related to the terpenoid biosynthesis pathway to compensate for the metabolic deficiency.

#### 3.3.3. Biosynthesis of Amino Acids

The metabolomic data showed that many metabolites with high *p*-values were enriched in amino acid synthesis and metabolism, and the transcriptomic data also showed that there were many differentially expressed genes related to amino acids. However, we do not think that this has any effect on the growth of *Chlamydomonas reinhardtii*. Our detection of soluble proteins (Figure 4g) showed that there was no significant difference in the total protein content of the WT, C18 and P1. We think that overexpression of the *BKT* gene does not have a big impact on the obtained mutants, and the excessive differences in amino acid synthesis and metabolism are due to the physiological changes in *Chlamydomonas reinhardtii* itself. The increase in carotenoids may affect the balance of amino acid synthesis and metabolism in *Chlamydomonas reinhardtii*, because they consume the precursors of amino acids, and also affect the microalgae’s response to light, temperature, nitrogen and other environmental factors. Therefore, the increase in carotenoids may have complex and multifaceted effects on amino acid synthesis and metabolism in *Chlamydomonas reinhardtii*.

#### 3.3.4. Glycolysis and Unsaturated Fatty Acids

In *Chlamydomonas reinhardtii*, an increase in carotenoid content can promote sugar metabolism. Studies have shown that cells can regulate lipid and carotenoid metabolism to cope with environmental changes [34,35]. Under heterotrophic conditions, lipid content increased by about 1.5-fold and β-carotene content increased by about 3-fold in *Chlamydomonas reinhardtii* [36]. Another study found that interfering with the activity of starch branching enzyme (DBE) via genetic engineering methods can increase lipid and carotenoid accumulation in *Chlamydomonas reinhardtii* under high-light conditions [37]. This is because DBE interference can promote carbon resource transfer from starch to lipids and carotenoids. Some studies have found that carotenoid and sugar contents are positively correlated in some plants [38]. For example, in tomatoes, high-carotenoid varieties also have high sugar content [31]. In kale, carotenoid accumulation is associated with an increase in soluble sugars in leaves [39]. In summary, in *Chlamydomonas reinhardtii*, carotenoids are not only important pigments and antioxidants, but also regulators of sugar metabolism and carbon allocation.

**Figure 14:**
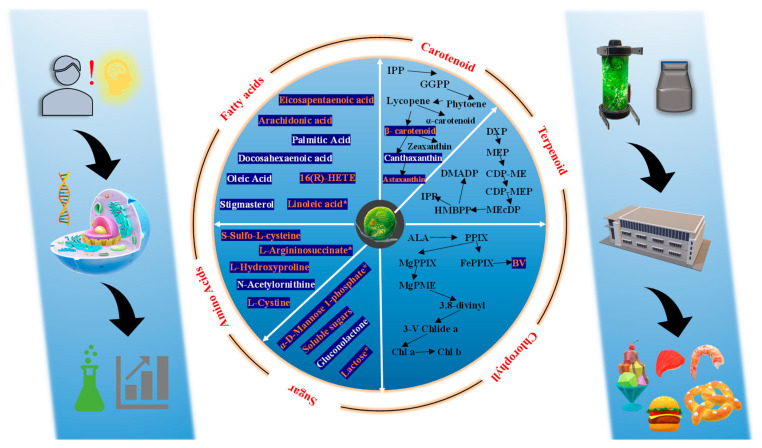
Metabolic pathway changes in *PrBKT* and *CrBKT* overexpression strains C18 and P1 vs. WT. The orange font with the blue background represents the compound up-regulated and the white font with the blue background represents the compound down-regulated. Compounds with * indicate that the differential compound was detected only in P1. Data were derived from the biochemical experiments of this experiment and the log^2^ FC (differential fold change of metabolites in different groups) of the metabolome. The red font on the outermost circle represents that most of the gene expression levels of this pathway were up-regulated in C18 and P1; the data were derived from the transcriptome. IPP: isopentenyl diphosphate, GGPP: geranylgeranyl diphosphate, DXP: 1-deoxy-D-xylulose 5-phosphate, MEP: 2-C-methyl-D-erythritol 4-phosphate, CDP-ME: 2- phospho- 4-(cytidine 5′-diphospho)-2-C- methyl-D- erythritol, CDP-MEP: 2-phospho-4-(cytidine 5′-diphospho)-2-C-methyl-D-erythritol, MEcDP: 2-C-methyl-D-erythritol 2,4-cyclodiphosphate, HMBPP: 4-hydroxy-3-methylbut-2-enyl pyrophosphate, DMADP: dimethylallyl diphosphate, ALA: 5-aminolevulinic acid, PPIX: protoporphyrin IX, MgPPIX: magnesium protoporphyrin, FePPIX: iron protoporphyrin IX complex, BV: biliverdin, MgPME: magnesium protoporphyrin IX monomethyl ester, 3,8-divinyl: 3,8-divinyl protochlorophyllide, 3-V Chlide a: 3-ethenylchlorophyllide a, Chl a: chlorophyll a, Chl b: chlorophyll b, 16(R)-HETE: 16R-hydroxy-5Z,8Z,11Z,14Z-eicosatetraenoic acid.

Therefore, in this study, we hypothesized that the increase in carotenoid content caused more carbon to be allocated to sugar metabolism, which was also consistent with the transcriptomic and metabolomic data. Most genes of the glycolysis and pentose phosphate pathway were up-regulated, and soluble sugars were measured to find that C18 and P1 had higher soluble sugar content than did the WT (Figure 4h). Additionally, in *Chlamydomonas reinhardtii*, the regulation of sugar and unsaturated fatty acids in the heterotrophic mode seems to be positively correlated (efficient heterotrophic cultivation of *Chlamydomonas reinhardtii*), especially under heterotrophic culture where cells do not need to perform photosynthesis and have more carbon available (Figure 14).

### 3.4. The Combination of Genetic Engineering and Heterotrophy for the Commercialization of Chlamydomonas reinhardtii

The production of microalgal carotenoids has been a hot topic in recent years, and the common targets of genetic engineering are *Haematococcus pluvialis*, *Dunaliella salina*, *Chlorella vulgaris* and others [40]. Compared with traditional plant carotenoid production, natural microalgal carotenoids have short production cycles, low costs, and high sustainability [38,41]. In this study, we combined the heterotrophic cultivation of *Chlamydomonas reinhardtii* with genetic engineering in the hope of obtaining some novel data.

The advantages of the heterotrophic cultivation of microalgae have been well-known due to the recent years of research [42]. Compared with open cultivation, heterotrophic fermentation in industrialization can obtain a high density of microalgal cultures, and the whole process is safe and controllable [43]. Since the fermentation environment can be controlled via modern industrial integration, the culture conditions can be precisely adjusted to increase the biomass and achieve better culture outcomes. In addition, the closed system of the fermenter can also effectively avoid external contamination and ensure the safety of the culture [44].

**Figure 15:**
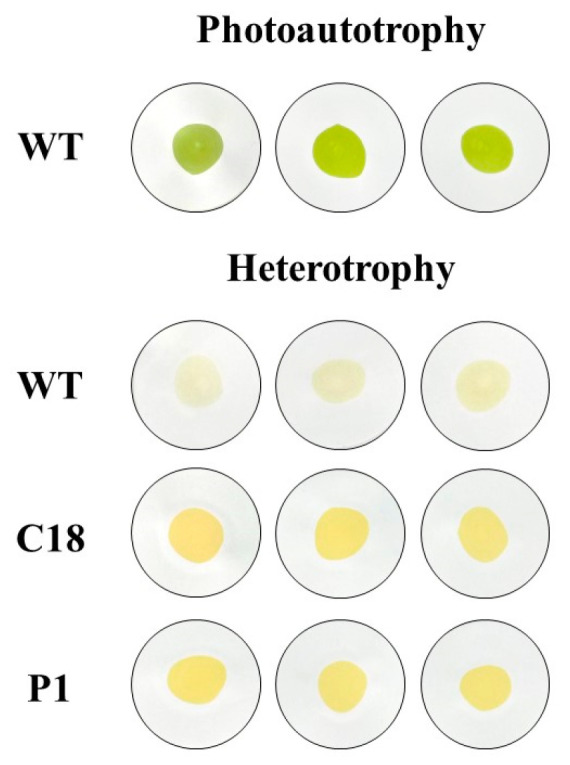
Colors of dried cells of the WT, C18 and P1. For each picture, 5 mL of *Chlamydomonas reinhardtii* cells were filtered through 0.22 μm membrane and dried.

With ordinary heterotrophy, in as little as 6–7 days, the cell density of *C. reinhardtii* will reach more than 7–8 × 10^6^ cell/mL. If a high-density culture is performed, the cell density will exceed 9 × 10^6^ cell/mL or more. Even in the absence of other high-value metabolites, the algal contribution to protein yield from *Chlamydomonas reinhardtii* would be substantial, whereas *Chlamydomonas reinhardtii* subjected to our overexpression had >2-fold higher total carotenoid production than the did wild type. Additionally, the overexpression strains C18 and P1 were yellow after heterotrophic treatment (Figure 15). We believe that this color will be more easily accepted by consumers for the strains as a food supplement. Additionally, combined with heterotrophic appreciable biomass, the commercial development of *C. reinhardtii* would even further favored if further modifications to the *BKT* gene are performed or if it is a multigene combined overexpression.

## 4. Materials and Methods

### 4.1. Culture Conditions of Chlamydomonas reinhardtii

The *Chlamydomonas reinhardtii* strain used in this experiment was CC-125 (wild-type; mating type+) and was obtained from the Chlamydomonas Resource Center (CRC, University of Minnesota). CC-125 cells were continuously cultured in a standard TAP medium in 500 mL erlenmeyer flasks (containing 150 mL TAP medium) [45]. *Chlamydomonas reinhardtii* cells before electro transformation were mixed and cultured. The cultures were grown under a temperature of 25 °C with a light intensity of 80 μmol m^−2^.s^−1^, a 14:10 h (light:dark) photoperiod, and constant shaking at 150 rpm. *Chlamydomonas reinhardtii* after electro transformation was heterotrophically cultured with TAP-PA (TAP containing paromomycin). The cultures were grown under a temperature of 25 °C without light, and with constant shaking at 150 rpm. Both two cultural models were cultured in an illumination incubator with normal white LEDs (model number: GXZ, Ningbo Jiangnan Instrument Factory, Ningbo, China). The CC-125 strain was maintained on a 1.5% agar TAP plate under low light (30 μmol photos m^−2^.s^−1^) at 25 °C and refreshed every month.

For mixed cultivation and heterotrophic cultivation, the cultures were diluted every 5 days to maintain a cell concentration of about 1.0 × 10^6^ cell/mL, as well as to maintain a steady carbonate system. The culture conditions for *C. reinhardtii* recovery and the selection of colonies after transformation are described in Section 4.3.

### 4.2. Vector Construction

We used SnapGene 4.3.6 for vector design. The backbone of the vector is PBI221, purchased from Wuhan Miaoling Biotechnology Co., Ltd., Wuhan, China (http://www.miaolingbio.com/, accessed on 1 June 2023).

The process for obtaining the *BKT* gene fragment is described below. *Chlamydomonas reinhardtii* β-Carotenoid ketogenese (*CrBKT*) gene information was obtained from both the NCBI (National Center for Biotechnology Information, accessed on 16 January 2021.) database and the JGI (Joint Genome Institute) database. The JGI gene ID of *CrBKT* is cre04.g215000, and the NCBI gene ID of *CrBKT* is CHLRE_04g215000v5. Additionally, the *CrBKT* gene fragment added into PBI221 was derived from the CDS of *CrBKT* after codon optimization. Then, ‘Run blast (standard nucleotide blast)’ was performed in NCBI using *CrBKT* gene mRNA (NCBI reference sequence: xm_001698647.1) with the program for highly simple sequences (‘Megablast’), clicking on ‘Blast’, to start the alignment. The *PrBKT* gene of the yeast *Phaffia rhodozyma* with the highest homology (NCBI reference sequence: LN483254.1) was selected for the *PrBKT* gene fragment after codon optimization. Codon optimization was performed using the website https://www.novoprolabs.com/tools/codon-optimization, accessed on 18 January 2021. The sequence type was set to DNA and the optimization of species was set to *Chlamydomonas reinhardtii*.

The paromomycin resistance gene (PARA) sequence was maintained in *E. coli* by our group. The PARA fragment was added at the region between the EGFP (enhanced green fluorescent protein) and terminator, and a single cutting enzyme in the backbone was selected as the junction point. After codon optimization, the *CrBKT* and *PrBKT* gene fragments were added at the region between the promoter and EGFP in the PBI221, respectively. Then, we obtained two designed vectors, PBI221-*CrBKT* and PBI221-*PrBKT*. Information of the two vectors was sent to Nanjing Genscript Bio Technology Co., Ltd., Nanjing, China, where the PARA, *CrBKT* and *PrBKT* gene fragments were directly synthesized, enzymatically linked and verified via sequencing based on the designed digestion sites in the sent PBI221. Sequences of Vectors PBI221-*CrBKT* and PBI221-*PrBKT* are shown in Supplemental Material S1. Plasmid extraction involved the use of Tiangen (https://www.tiangen.com/) high-purity plasmid Mini Extraction Kit (DP104).

### 4.3. Transformation

CC-125 cells were electroporated using the gene Pluser Xcell Electroporation system. The conditions of electric rotation were optimized from our laboratory. For pretransformation, cells at the mid logarithmic growth phase (approximately 1–3 ^ 10^6^ cell/mL for cell number and 0.3 for OD_750_) were collected, centrifuged at 3000× *g* for 5 min at room temperature and then resuspended with TAP (containing 40 mM sucrose) to a cell density of 1 × 10^8^ cell mL^−1^. Next, these cells were shaken by a shaker at 250 rpm, for 30 min, at 40 °C. Briefly, 250 μL treated cultures and extracted plasmids (PBI221-*CrBKT* and PBI221-*PrBKT*) were added into a 4 mm electric cuvette (Bio-Rad) for transformation. The electrical transition parameters were 600 V, 50 μF, and infinity resistance. After transformation, cells were transferred to 10 mL of a TAP-40 mM solution with low light (30 μmol photos m^−2^.s^−1^), at 50 rpm for overnight recovery. Cells were spread evenly on TAP-PARA plates with low light (30 μmol photos m^−2^.s^−1^) after recovery.

### 4.4. Quantification of Overexpression in C. reinhardtii Colonies

One week after the procedure described in Section 4.3, single CC-125 colony strains were picked from the TAP-PA plates, and transferred into 96-well plates containing a liquid TAP-PA medium. After 4–5 days, normally growing colonies were selected and transferred to 24-well plates for an additional 4–5 days of culture, and EGFP expression status was assessed via inverted fluorescence microscopy at 488 nm. Colonies containing a normal EGFP signal were numbered and expanded in heterotrophic culture. The DNA and RNA of these colonies were extracted separately, and subjected to agarose gel electrophoresis and qRT-qPCR (Methods are detailed in Section 4.9).

### 4.5. Growth Measurement

In terms of densitometry, the cell density of *Chlamydomonas reinhardtii* was positively correlated with the OD_750_, which could be expressed via a detection of the OD_750_ of the cultures, after mixing 3–4 mL of the tested *Chlamydomonas reinhardtii* cultures and pouring the mixture into a cuvette to detect the absorbance value at 750 nm using an ultraviolet spectrophotometer.

In terms of cytometry, using dilutions supplied with the Beckman Coulter hematology analyzer, 1 mL of the tested cells was taken and diluted 20-fold (40-fold dilution in the logarithmic growth phase) for counting using the Beckman Coulter Z2 cytometer (https://www.beckmancoulter.cn/, accessed on 8 October 2020).

### 4.6. Carotenoid Extraction and Analysis

Briefly, 5 mL of *Chlamydomonas reinhardtii* cultures was centrifuged at 3000× *g* for 10 min and then to it was added 5 mL of an 80% acetone solution. After vortexing, the mixture was put in the dark overnight at 4 °C. On the next day, the mixture was centrifuged at 12,000× *g* and 4 °C for 10 min. The supernatant was taken and determined at OD_470_, OD_646_ and OD_663_ using an ultraviolet spectrophotometer. Computational methods of carotenoid contents refer to those in the published paper by Lichtenthaler et al. [46].

Carotenoid extracts were obtained using HPLC (High performance liquid chromatography–Agilent 1260 Infinity II) with a WondaSil^TM^ C18 column (5 μm) and with the absorbance at 475 nm (astaxanthin) and 450 nm (β-Carotenoid). Mobile phase A is MeOH, and mobile phase B is ACN. The mobile phase was eluted for a total of 30 min with a volume percentage of 55% for phase A and 45% for phase B. The injection volume was 10 μL, and the flow rate was 1 mL/min.

### 4.7. Processing of C. reinhardtii Colonies for Transcriptome and Metabolome

In this study, for the transcriptome and metabolome analysis, the CC-125 strains (mutation types) were cultured under heterotrophy until the logarithmic growth phase, and then were centrifuged at 3000× *g* for 10 min. We collected ~4 × 10^6^ cells of each group for transcriptome and ~ 2 × 10^7^ cells of each group for the metabolome. The cell numbers of each group counted each time when collected are shown in Supplementary Figure S2. The cells collected after centrifugation were rinsed with PBS three times, then transferred into liquid nitrogen for quick freezing, and transferred to a −80 °C refrigerator for storage overnight. The processed samples were sent to Novogene Co., Ltd., (Beijing, China) to complete the transcriptome and non-targeted metabolome (see Supplementary Materials S5 and S6 for detailed metabolome and transcriptome experimental methods).

### 4.8. Determination of Total Protein Content and Soluble Sugar Content

For total protein content and soluble sugar content measurement, we used the cells that were stored at −80 °C in a refrigerator. After adding PBS (per 5 × 10^6^ cells mL^−1^ with 1 mL of 1 × PBS), the *C. reinhardtii* cells were disrupted via sonication (power, 25%; sonication, 5 s; interval, 5 s; repeat 30 times). After centrifugation at 10,000× *g* and at 4 °C for 10 min, the supernatant was removed and kept on ice for testing. Then, the treated supernatant was detected using the following kits: plant soluble sugar content test kit A145-1-1 (Nanjing Jiancheng Bioengineering institute, Nanjing, China), and Bradford Protein Assay Kit PC0010 (Beijing Solarbio Technology Co., Ltd., Beijing, China).

### 4.9. Quantitative Real-Time Quantitative PCR (qRT-PCR)

RNA was extracted using Tiangen RNAprep Pure Plant Plus Kit (ploysaccharide and polyphenolic-rich). After the determination of the RNA quality via agarose gel electrophoresis, and complementary (c)DNA synthesis was carried out using ReverTra Ace qPCR RT Master Mix with gDNA Remover (TOYOBO, Osaka, Japan). Quantitative real-time-PCR (qRT-PCR) was performed using TB Green^®^ Premix Ex Taq™ II (Tli RNaseH Plus) (TaKaRa Bio Inc., Shiga, Japan) to measure the relative transcript levels of the genes associated with carotenoid metabolism. The gene encoding β-actin was used as the endogenous control. The genes and the primers of qRT-PCR are listed in the Supplementary Materials S4. Transcript levels were calculated using Graphpad prism 8 and Microsoft excel via the double difference method (double delta CT; ΔΔ Ct).

### 4.10. Statistical Analysis

The experiments were performed with three replicates for each treatment. The results of the biochemical and physiological parameter values obtained for all the variables were subjected to an analysis of variance (one-way ANOVA, *p* < 0.05) followed by Tukey’s test (*p* ≤ 0.05) using SPSS 20.0 software. And in figures, the same Minuscule mean no significant difference, while different Minuscule mean significant difference. The error bars in bar charts and line charts represent the standard deviation (SD) values for each group of data. Transcriptome data have been uploaded to NCBI (National Center for Biotechnology Information) (accession number: PRJNA940914). The results of the qRT-PCR were calculated as described above. PowerPoint and GraphPad Prism 8 were used to draw and modify the graphics.

## 5. Conclusions

In conclusion, we have successfully enhanced carotenoid production in *Chlamydomonas reinhardtii* by overexpressing two different *BKT* genes from *Phaffia rhodozyma* and *Chlamydomonas reinhardtii*. We have demonstrated that both *BKT* genes can increase the levels of β-carotene and astaxanthin in the transformed alga strains, and that *PrBKT* has a broader effect on the carotenoid pathway than doe *CrBKT*. We also revealed via transcriptome and metabolome analyses that endogenous and exogenous *BKT* gene overexpression led to changes in some key genes involved in the regulation of carotenoid biosynthesis and canthaxanthin. Our results provide new insights into microalgal carotenoid synthesis using genetic engineering.

## Figures and Tables

**Table 1:** Summary of transcriptome sequencing data quality.

Sample	Raw Reads ^1^	Raw Bases ^2^	Clean Reads ^3^	Clean Bases ^4^	Error Rate ^5^	Q30 (%) ^6^	GC Pct ^7^	Total Map Pct ^8^	Unique Map ^9^
WT-1	45,897,466	6.88 G	43,481,436	6.52 G	0.03	90.57	62.75	95.04%	92.66%
WT-2	45,372,590	6.81 G	43,300,230	6.5 G	0.03	90.04	62.97	95.08%	92.72%
WT-3	47,243,698	7.09 G	45,441,558	6.82 G	0.03	90.75	62.68	94.98%	92.53%
C18-1	45,981,798	6.9 G	43,321,074	6.5 G	0.03	90.29	61.28	87.64%	83.55%
C18-2	47,989,038	7.2 G	45,152,476	6.77 G	0.03	90.53	62.04	90.40%	87.63%
C18-3	49,309,270	7.4 G	46,452,078	6.97 G	0.03	89.37	63.72	93.53%	90.35%
P1-1	51,821,018	7.77 G	48,753,422	7.31 G	0.03	92.94	61.93	87.88%	84.91%
P1-2	48,975,588	7.35 G	46,310,808	6.95 G	0.03	93.22	62.65	89.23%	86.45%
P1-3	42,490,208	6.37 G	39,192,990	5.88 G	0.03	91.93	61.33	82.86%	80.43%

^1^ Raw reads: number of reads in raw data; ^2^ raw bases: number of bases of the raw data (raw base = raw reads × 150 bp); ^3^ clean reads: number of reads after raw data filtering; ^4^ clean bases: raw data filtered bases (clean base = clean reads × 150 bp); ^5^ error rate: data overall sequencing error rate; ^6^ Q30 (%): percentage of total bases with phred values greater than 30; ^7^ GC pct: percentage of G and C bases in the four bases in clean reads; ^8^ Total map: percentage of clean reads aligned to the genome; ^9^ percentage of clean reads aligned to unique positions of the reference genome (for subsequent quantitative data analysis of reads).

**Table 2:** Differential screening results for metabolites.

Compared Samples ^1^	Num. of Total Ident. ^2^	Num. of Total Sig. ^3^	Num. of Sig.Up ^4^	Num. of Sig.Down ^5^
C18 vs. WT_pos *	595	240	68	172
C18 vs. WT_neg ^#^	335	136	40	96
P1 vs. WT_pos	595	222	117	105
P1 vs. WT_neg	335	113	60	53

^1^ Compared samples: sample pairs compared, for the former compared to the latter; ^2^ Num. of Total Ident.: total metabolite identification results; ^3^ Num. of Total Sig.: total number of significantly differentially expressed metabolites; ^4^ Num. of Sig.Up: total number of significantly up-regulated metabolites; ^5^ Num. of Sig.Down: total number of significantly down-regulated metabolites; * pos: positive ion; ^#^ neg: negative ion.

## Data Availability

The authors confirm that the data supporting the findings of this study are available within the article and its Supplementary Materials.

## Supplementary Materials

The following supporting information can be downloaded at: https://www.mdpi.com/article/10.3390/ijms241411382/s1. References [47,48,49,50,51,52] are cited in the supplementary materials.
