# Supplementary material for: Enhanced Carotenoid Production in Chlamydomonas reinhardtii by Overexpression of Endogenousand Exogenous Beta-Carotene Ketolase (BKT) Genes
Source: Int J Mol Sci. 2023 Jul 13;24(14):11382. doi: 10.3390/ijms241411382 (PMC10379168; doi:10.3390/ijms241411382)
Supplement: Supplementary file 1 [file ijms-24-11382-s001.zip › Cutin, suberine and wax biosynthesis.pdf]

CUTIN, SUBERINE AND WAX BIOSYNTHESIS

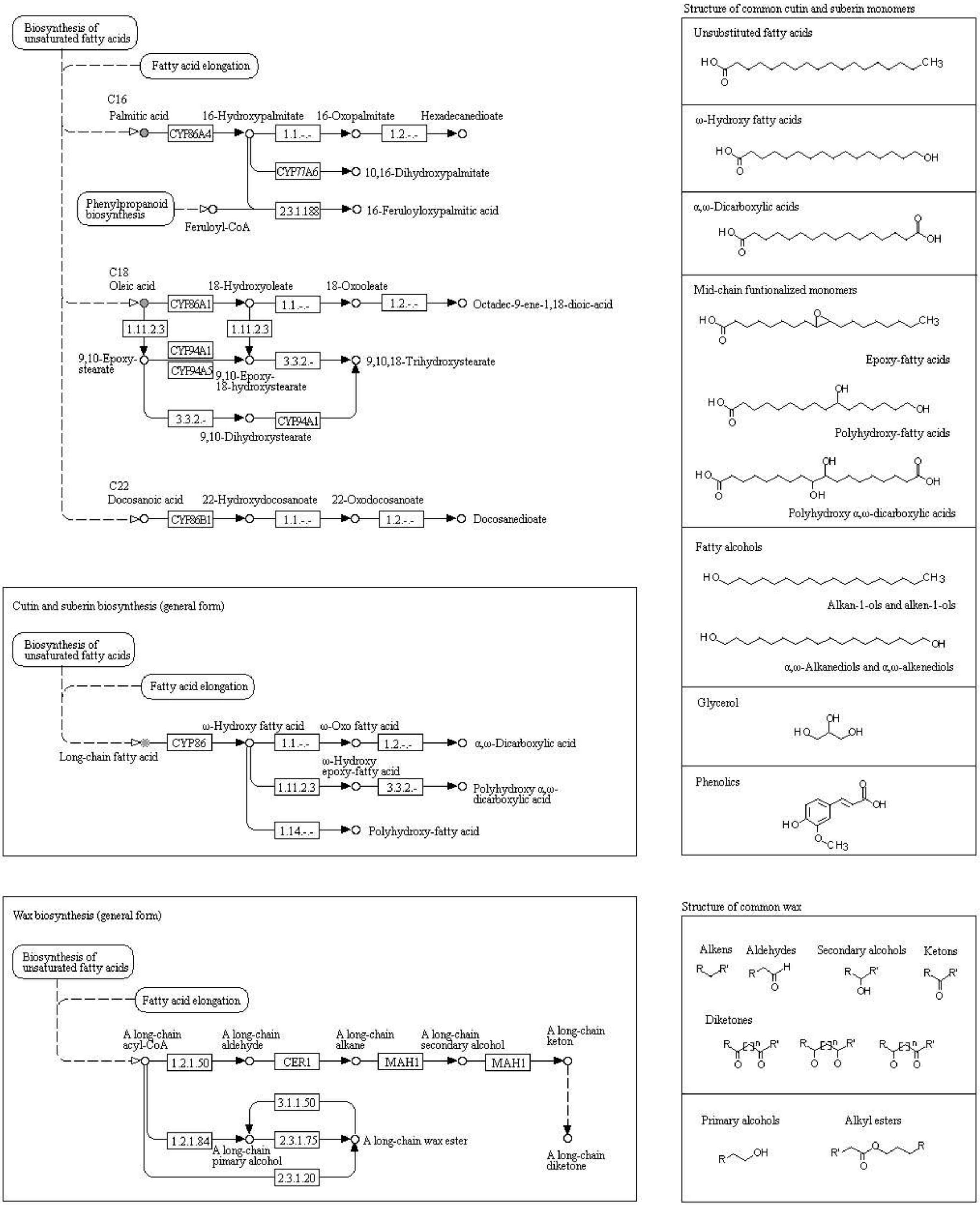

00073 3/14/17
(c) Kanehisa Laboratories
