# Supplementary material for: Enhanced Carotenoid Production in Chlamydomonas reinhardtii by Overexpression of Endogenousand Exogenous Beta-Carotene Ketolase (BKT) Genes
Source: Int J Mol Sci. 2023 Jul 13;24(14):11382. doi: 10.3390/ijms241411382 (PMC10379168; doi:10.3390/ijms241411382)
Supplement: Supplementary file 1 [file ijms-24-11382-s001.zip › Supplementary figures and materials.pdf]

Supplemental Figures and Materials

Supplemental Figure S1. Agarose gel electrophoresis for PARA

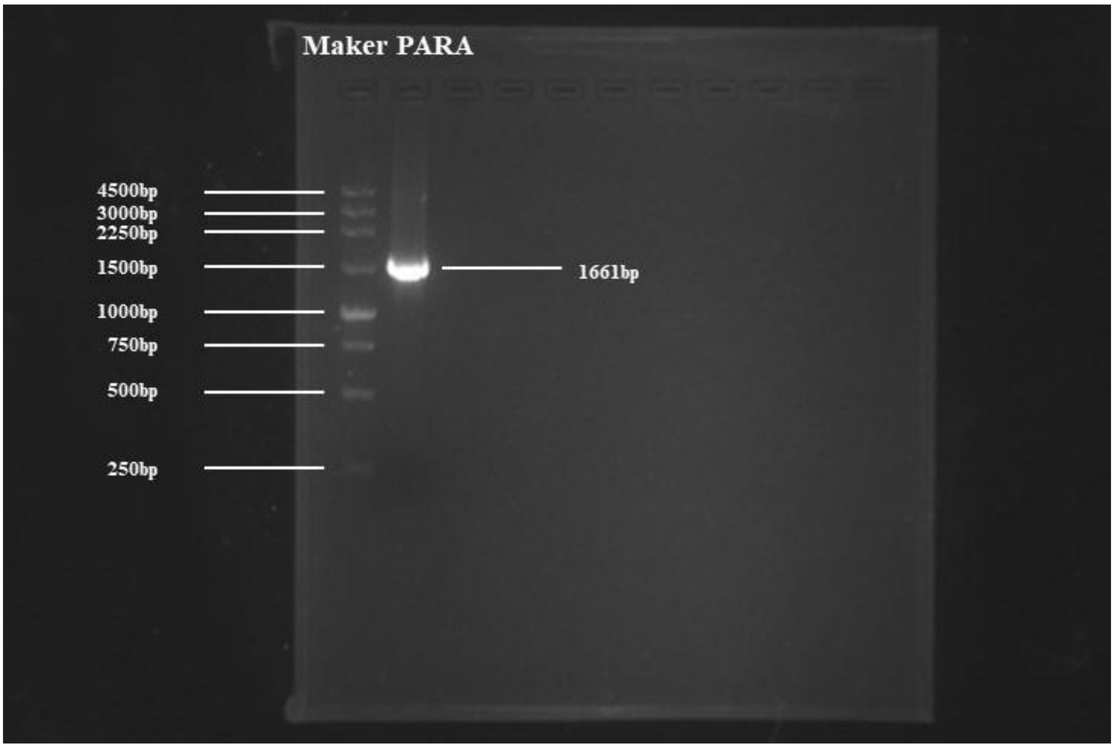

Supplementary Figure S2. Cell numbers at the time of sample collection for the transcriptome and metabolome

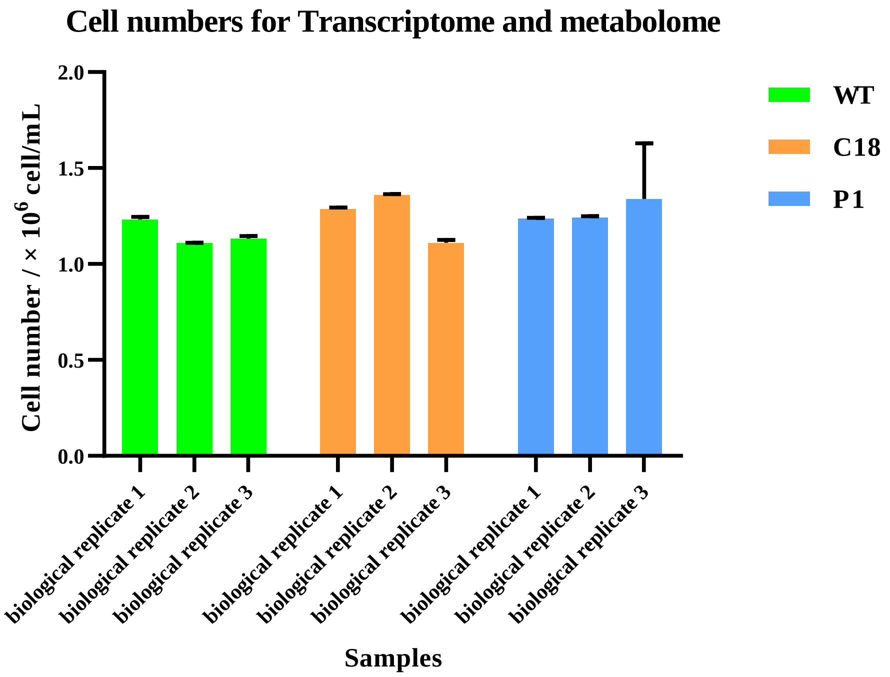

Supplementary material S1. Sequence of the vector PBI221-CrBKT and PBI221-PrBKT

PBI221-CrBKT

AAGCTTGCATGCCTGCAGGTCCCCAGATTAGCCTTTTCAATTTCAGAAAGAATGCTAACCCACAGATG
GTTAGAGAGGCTTACGCAGCAGGTCTCATCAAGACGATCTACCCGAGCAATAATCTCCAGGAAATCAA
ATACCTTCCCAAGAAGGTTAAAGATGCAGTCAAAAGATTCAGGACTAACTGCATCAAGAACACAGAG
AAAGATATATTTCTCAAGATCAGAAGTACTATTCCAGTATGGACGATTCAAGGCTTGCTTCACAAACCA
AGGCAAGTAATAGAGATTGGAGTCTCTAAAAAGGTAGTTCCCACTGAATCAAAGGCCATGGAGTCAA
AGATTCAAATAGAGGACCTAACAGAACTCGCCGTAAAGACTGGCGAACAGTTCATACAGAGTCTCTTA
CGACTCAATGACAAGAAGAAAATCTTCGTCAACATGGTGGAGCACGACACACTTGTCTACTCCAAAA
ATATCAAAGATACAGTCTCAGAAGACCAAAGGGCAATTGAGACTTTTCAACAAAGGGTAATATCCGGA
AACCTCCTCGGATTCCATTGCCCAGCTATCTGTCACTTTATTGTGAAGATAGTGGAAAAGGAAGGTGGC
TCCTACAAATGCCATCATTGCGATAAAGGAAAGGCCATCGTTGAAGATGCCTCTGCCGACAGTGGTCC
CAAAGATGGACCCCCACCCACGAGGAGCATCGTGGAAAAAGAAGACGTTCCAACCACGTCTTCAAA
GCAAGTGGATTGATGTGATATCTCCACTGACGTAAGGGATGACGCACAATCCCACTATCCTTCGCAAGA
CCCTTCCTCTATATAAGGAAGTTCATTTCATTTGGAGAGAACACGGGGGACTCTAGAATGGCCGTCATG
ATGCGCACCCAGGCGCCCGCTGCCACTCGCGCTTCATCGCGCGTCGCTGTTGCCGCTCGCCCGGCTGC
TCGCCGCGCCGTGGTGGTCCGCGCCGAGGCTGAGGCTGCCCCTGCTGCTGCCAAGAAGGCTGCTGAG
AAGCCCGCCTGGACTGTGCCGACCCTGAACCCCGACACTCCCAGCCCGATTTTCGGTGGCAGCACCG
GCGGTCTGCTGCGCAAGGCTCAGACTGAGGAGTTCTACGTCATCACCTGGGAGGCTAAGAAGGAGCA
GATCTTCGAGATGCCCACTGGCGGTGCCGCTATCATGCGCCAGGGCCCCAACCTGCTGAAGTTCGGCA
AGAAGGAGCAGTGCCTCGCCCTGACGACCCAGCTCCGCAACAAGTTCAAGCTGACCCCCTGCTTCTA
CCGCGTCTTCCCCGACGGCAAGGTGCAGTACCTGCACCCTGCTGACGGCGTCTACCCCGAGAAGGTG
AACGCTGGCCGCGTGGGCGCGAACCAGAACATGCGCCGCATCGGCCAGAACGTCAACCCCATCAAGG
TCAAGTTCTCTGGCCGCATGATGTCGCCTGCTGAGATCGGAGGAGGAGGAGGATCCGAATTCGAGCTC
ATGGGCCCTGGAATCCAGCCCACCTCGGCACGGCCGTGCAGCAGGACCAAGCATTCCCGCTTCGCGC
TCCTCGCTGCCGCACTCACCGCGCGCAGGGTGAAGCAATTCACGAAGCAGTTCCGAAGCCGGCGCAT
GGCGGAGGACATCTTAAAACTGTGGCAGCGGCAGTACCACCTTCCCCGCGAGGACAGCGACAAGCGC
ACACTGCGGGAGCGCGTGCACCTGTACCGCCCCCCACGGTCGGACCTGGGTGGCATTGCGGTCGCGG
TGACGGTCATAGCCCTGTGGGCAACCCTGTTCGTGTATGGGCTGTGGTTCGTCAAGCTGCCGTGGGCG
CTCAAGGTGGGCGAGACAGCCACCAGCTGGGCAACGATCGCTGCCGTTTTTTTCTCGCTGGAGTTCCT
GTACACAGGGCTGTTCATCACCACGCACGACGCCATGCACGGCACTATTGCGCTGCGCAACCGCAGGC
TTAACGACTTCCTGGGCCAGCTGGCCATCAGCCTGTATGCGTGGTTTGATTACTCTGTGCTGCACCGCA
AGCATTGGGAGCACCACAACCACACGGGTGAACCGAGAGTGGACCCCGATTTCCACCGTGGCAACCC
CAACCTGGCGGTGTGGTTCGCGCAGTTCATGGTGTCCTACATGACGCTGTCCCAGTTTCTGAAGATCG
CGGTATGGAGCAATTTGTTGCTACTGGCCGGCGCGCCGCTGGCCAACCAGCTGCTGTTCATGACCGCA
GCGCCCATCCTGTCGGCGTTCCGGCTCTTCTACTACGGCACCTACGTGCCGCACCACCCGGAGAAGGG
CCACACGGGGGCCATGCCGTGGCAGGTGTCACGTACTTCCAGCGCTAGTCGGCTGCAGAGCTTTCTTA
CCTGCTACCACTTCGACCTGCACTGGGAGCACCATCGCTGGCCGTACGCGCCTTGGTGGGAGCTGCCC
AAGTGCCGCCAGATCGCCCGCGGCGCCGCTTTGGCCCCAGTCGACAAGCTTATGGTGAGCAAGGGCG
AGGAGCTGTTCACCGGGGTGGTGCCCATCCTGGTCGAGCTGGACGGCGACGTAAACGGCCACAAGTT
CAGCGTGTCCGGCGAGGGCGAGGGCGATGCCACCTACGGCAAGCTGACCCTGAAGTTCATCTGCACC
ACCGGCAAGCTGCCCGTGCCCTGGCCCACCCTCGTGACCACCTTCACCTACGGCGTGCAGTGCTTCAG
CCGCTACCCCGACCACATGAAGCAGCACGACTTCTTCAAGTCCGCCATGCCCGAAGGCTACGTCCAGG
AGCGCACCATCTTCTTCAAGGACGACGGCAACTACAAGACCCGCGCCGAGGTGAAGTTCGAGGGCGA

CACCCTGGTGAACCGCATCGAGCTGAAGGGCATCGACTTCAAGGAGGACGGCAACATCCTGGGGCAC
AAGCTGGAGTACAACTACAACAGCCACAACGTCTATATCATGGCCGACAAGCAGAAGAACGGCATCA
AGGTGAACTTCAAGATCCGCCACAACATCGAGGACGGCAGCGTGCAGCTCGCCGACCACTACCAGCA
GAACACCCCCATCGGCGACGGCCCCGTGCTGCTGCCCGACAACCACTACCTGAGCACCCAGTCCGCC
CTGAGCAAAGACCCCAACGAGAAGCGCGATCACATGGTCCTGCTGGAGTTCGTGACCGCCGCCGGGA
TCACTCACGGCATGGACGAGCTGTACAAGTAAAGCGGCCCGAATTTCCCCGATCGTTCAAACATTTGG
CAATAAAGTTTCTTAAGATTGAATCCTGTTGCCGGTCTTGCGATGATTATCATATAATTTCTGTTGAATTA
CGTTAAGCATGTAATAATTAACATGTAATGCATGACGTTATTTATGAGATGGGTTTTTATGATTAGAGTCC
CGCAATTATACATTTAATACGCGATAGAAAACAAAATATAGCGCGCAAACTAGGATAAATTATCGCGCG
CGGTGTCATCTATGTTACTAGATCGGGAATTCACTGGCCGTCGTTTTACAACGTCGTGACTGGGAAAAC
CCTGGCGTTACCCAACTTAATCGCCTTGCAGCACATCCCCCTTTCGCCAGCTGGCGTAATAGCGAAGA
GGCCCGCACCGATCGCCCTTCCCAACAGTTGCGCAGCCTGAATGGCGAATGGCGCCTGATGCGGTATT
TTCTCCTTACGCATCTGTGCGGTATTTCACACCGCATATGAGCTTGCATGCCGGGCGCGCCAGAAGGAG
CGCAGCCAAACCAGGATGATGTTTGATGGGGTATTTGAGCACTTGCAACCCTTATCCGGAAGCCCCCT
GGCCCACAAAGGCTAGGCGCCAATGCAAGCAGTTCGCATGCAGCCCCTGGAGCGGTGCCCTCCTGAT
AAACCGGCCAGGGGGCCTATGTTCTTTACTTTTTTACAAGAGAAGTCACTCAACATCTTAAAATGGCC
AGGTGAGTCGACGAGCAAGCCCGGCGGATCAGGCAGCGTGCTTGCAGATTTGACTTGCAACGCCCGC
ATTGTGTCGACGAAGGCTTTTGGCTCCTCTGTCGCTGTCTCAAGCAGCATCTAACCCTGCGTCGCCGTT
TCCATTTGCAGGATGCTCGAGATTCGAAGCATGGACGATGCGTTGCGTGCACTGCGGGGTCGGTATCC
CGGTTGTGAGTGGGTTGTTGTGGAGGATGGGGCCTCGGGGGCTGGTGTTTATCGGCTTCGGGGTGGTG
GGCGGGAGTTGTTTGTCAAGGTGGCAGCTCTGGGGGCCGGGGTGGGCTTGTTGGGTGAGGCTGAGCG
GCTGGTGTGGTTGGCGGAGGTGGGGATTCCCGTACCTCGTGTTGTGGAGGGTGGTGGGGACGAGAGG
GTCGCCTGGTTGGTCACCGAAGCGGTTCCGGGGCGTCCGGCCAGTGCGCGGTGGCCGCGGGAGCAGC
GGCTGGACGTGGCGGTGGCGCTCGCGGGGCTCGCTCGTTCGCTGCACGCGCTGGACTGGGAGCGGTG
TCCGTTCGATCGCAGTCTCGCGGTGACGGTGCCGCAGGCGGCCCGTGCTGTCGCTGAAGGGAGCGTC
GACTTGGAGGATCTGGACGAGGAGCGGAAGGGGTGGTCGGGGGAGCGGCTTCTCGCCGAGCTGGAG
CGGACTCGGCCTGCGGACGAGGATCTGGCGGTTTGCCACGGTGACCTGTGCCCGGACAACGTGCTGC
TCGACCCTCGTACCTGCGAGGTGACCGGGCTGATCGACGTGGGGCGGGTCGGCCGTGCGGACCGGCA
CTCCGATCTCGCGCTGGTGCTGCGCGAGCTGGCCCACGAGGAGGACCCGTGGTTCGGGCCGGAGTGT
TCCGCGGCGTTCCTGCGGGAGTACGGGCGCGGGTGGGATGGGGCGGTATCGGAGGAAAAGCTGGCGT
TTTACCGGCTGTTGGACGAGTTCTTCTGACATATGGTGCACTCTCAGTACAATCTGCTCTGATGCCGCA
TAGTTAAGCCAGCCCCGACACCCGCCAACACCCGCTGACGCGCCCTGACGGGCTTGTCTGCTCCCGG
CATCCGCTTACAGACAAGCTGTGACCGTCTCCGGGAGCTGCATGTGTCAGAGGTTTTCACCGTCATCA
CCGAAACGCGCGAGACGAAAGGGCCTCGTGATACGCCTATTTTTATAGGTTAATGTCATGATAATAATG
GTTTCTTAGACGTCAGGTGGCACTTTTCGGGGAAATGTGCGCGGAACCCCTATTTGTTTATTTTTCTAA
ATACATTCAAATATGTATCCGCTCATGAGACAATAACCCTGATAAATGCTTCAATAATATTGAAAAAGGA
AGAGTATGAGTATTCAACATTTCCGTGTCGCCCTTATTCCCTTTTTTGCGGCATTTTGCCTTCCTGTTTTT
GCTCACCCAGAAACGCTGGTGAAAGTAAAAGATGCTGAAGATCAGTTGGGTGCACGAGTGGGTTACA
TCGAACTGGATCTCAACAGCGGTAAGATCCTTGAGAGTTTTCGCCCCGAAGAACGTTTTCCAATGATG
AGCACTTTTAAAGTTCTGCTATGTGGCGCGGTATTATCCCGTATTGACGCCGGGCAAGAGCAACTCGGT
CGCCGCATACACTATTCTCAGAATGACTTGGTTGAGTACTCACCAGTCACAGAAAAGCATCTTACGGAT
GGCATGACAGTAAGAGAATTATGCAGTGCTGCCATAACCATGAGTGATAACACTGCGGCCAACTTACT
TCTGACAACGATCGGAGGACCGAAGGAGCTAACCGCTTTTTTGCACAACATGGGGGATCATGTAACTC
GCCTTGATCGTTGGGAACCGGAGCTGAATGAAGCCATACCAAACGACGAGCGTGACACCACGATGCC

TGTAGCAATGGCAACAACGTTGCGCAAACTATTAACTGGCGAACTACTTACTCTAGCTTCCCGGCAAC
AATTAATAGACTGGATGGAGGCGGATAAAGTTGCAGGACCACTTCTGCGCTCGGCCCTTCCGGCTGGC
TGGTTTATTGCTGATAAATCTGGAGCCGGTGAGCGTGGGTCTCGCGGTATCATTGCAGCACTGGGGCC
AGATGGTAAGCCCTCCCGTATCGTAGTTATCTACACGACGGGGAGTCAGGCAACTATGGATGAACGAA
ATAGACAGATCGCTGAGATAGGTGCCTCACTGATTAAGCATTGGTAACTGTCAGACCAAGTTTACTCAT
ATATACTTTAGATTGATTTAAAACTTCATTTTTAATTTAAAAGGATCTAGGTGAAGATCCTTTTTGATAAT
CTCATGACCAAAATCCCTTAACGTGAGTTTTCGTTCCACTGAGCGTCAGACCCCGTAGAAAAGATCAA
AGGATCTTCTTGAGATCCTTTTTTTCTGCGCGTAATCTGCTGCTTGCAAACAAAAAAACCACCGCTACC
AGCGGTGGTTTGTTTGCCGGATCAAGAGCTACCAACTCTTTTTCCGAAGGTAACTGGCTTCAGCAGAG
CGCAGATACCAAATACTGTCCTTCTAGTGTAGCCGTAGTTAGGCCACCACTTCAAGAACTCTGTAGCAC
CGCCTACATACCTCGCTCTGCTAATCCTGTTACCAGTGGCTGCTGCCAGTGGCGATAAGTCGTGTCTTA
CCGGGTTGGACTCAAGACGATAGTTACCGGATAAGGCGCAGCGGTCGGGCTGAACGGGGGGTTCGTG
CACACAGCCCAGCTTGGAGCGAACGACCTACACCGAACTGAGATACCTACAGCGTGAGCTATGAGAA
AGCGCCACGCTTCCCGAAGGGAGAAAGGCGGACAGGTATCCGGTAAGCGGCAGGGTCGGAACAGGA
GAGCGCACGAGGGAGCTTCCAGGGGGAAACGCCTGGTATCTTTATAGTCCTGTCGGGTTTCGCCACCT
CTGACTTGAGCGTCGATTTTTGTGATGCTCGTCAGGGGGGCGGAGCCTATGGAAAAACGCCAGCAAC
GCGGCCTTTTTACGGTTCCTGGCCTTTTGCTGGCCTTTTGCTCACATGTTCTTTCCTGCGTTATCCCCTG
ATTCTGTGGATAACCGTATTACCGCCTTTGAGTGAGCTGATACCGCTCGCCGCAGCCGAACGACCGAG
CGCAGCGAGTCAGTGAGCGAGGAAGCGGAAGAGCGCCCAATACGCAAACCGCCTCTCCCCGCGCGT
TGGCCGATTCATTAATGCAGCTGGCACGACAGGTTTCCCGACTGGAAAGCGGGCAGTGAGCGCAACG
CAATTAATGTGAGTTAGCTCACTCATTAGGCACCCCAGGCTTTACACTTTATGCTTCCGGCTCGTATGTT
GTGTGGAATTGTGAGCGGATAACAATTTCACACAGGAAACAGCTATGACCATGATTACGCC

PBI221-PrBKT:

AAGCTTGCATGCCTGCAGGTCCCCAGATTAGCCTTTTCAATTTCAGAAAGAATGCTAACCCACAGATG
GTTAGAGAGGCTTACGCAGCAGGTCTCATCAAGACGATCTACCCGAGCAATAATCTCCAGGAAATCAA
ATACCTTCCCAAGAAGGTTAAAGATGCAGTCAAAAGATTCAGGACTAACTGCATCAAGAACACAGAG
AAAGATATATTTCTCAAGATCAGAAGTACTATTCCAGTATGGACGATTCAAGGCTTGCTTCACAAACCA
AGGCAAGTAATAGAGATTGGAGTCTCTAAAAAGGTAGTTCCCACTGAATCAAAGGCCATGGAGTCAA
AGATTCAAATAGAGGACCTAACAGAACTCGCCGTAAAGACTGGCGAACAGTTCATACAGAGTCTCTTA
CGACTCAATGACAAGAAGAAAATCTTCGTCAACATGGTGGAGCACGACACACTTGTCTACTCCAAAA
ATATCAAAGATACAGTCTCAGAAGACCAAAGGGCAATTGAGACTTTTCAACAAAGGGTAATATCCGGA
AACCTCCTCGGATTCCATTGCCCAGCTATCTGTCACTTTATTGTGAAGATAGTGGAAAAGGAAGGTGGC
TCCTACAAATGCCATCATTGCGATAAAGGAAAGGCCATCGTTGAAGATGCCTCTGCCGACAGTGGTCC
CAAAGATGGACCCCCACCCACGAGGAGCATCGTGGAAAAAGAAGACGTTCCAACCACGTCTTCAAA
GCAAGTGGATTGATGTGATATCTCCACTGACGTAAGGGATGACGCACAATCCCACTATCCTTCGCAAGA
CCCTTCCTCTATATAAGGAAGTTCATTTCATTTGGAGAGAACACGGGGGACTCTAGAATGGCCGTCATG
ATGCGCACCCAGGCGCCCGCTGCCACTCGCGCTTCATCGCGCGTCGCTGTTGCCGCTCGCCCGGCTGC
TCGCCGCGCCGTGGTGGTCCGCGCCGAGGCTGAGGCTGCCCCTGCTGCTGCCAAGAAGGCTGCTGAG
AAGCCCGCCTGGACTGTGCCGACCCTGAACCCCGACACTCCCAGCCCGATTTTCGGTGGCAGCACCG
GCGGTCTGCTGCGCAAGGCTCAGACTGAGGAGTTCTACGTCATCACCTGGGAGGCTAAGAAGGAGCA
GATCTTCGAGATGCCCACTGGCGGTGCCGCTATCATGCGCCAGGGCCCCAACCTGCTGAAGTTCGGCA
AGAAGGAGCAGTGCCTCGCCCTGACGACCCAGCTCCGCAACAAGTTCAAGCTGACCCCCTGCTTCTA
CCGCGTCTTCCCCGACGGCAAGGTGCAGTACCTGCACCCTGCTGACGGCGTCTACCCCGAGAAGGTG

AACGCTGGCCGCGTGGGCGCGAACCAGAACATGCGCCGCATCGGCCAGAACGTCAACCCCATCAAGG
TCAAGTTCTCTGGCCGCATGATGTCGCCTGCTGAGATCGGAGGAGGAGGATCCGAATTCGAGCTCATG
GGTCCGGGTATCCAACCTACGAGCGCTCGCCCTTGCTCCCGCACCAAGCACAGCCGCTTCGCCCTGCT
GGCCGCGGCTCTGACCGCCCGCCGCGTCAAGCAGTTCACGAAGCAGTTCCGGTCGCGCCGGATGGCC
GAGGACATTCTCAAGCTGTGGCAGCGGCAGTACCACCTCCCGCGCGAGGACTCGGACAAGCGCACGC
TGCGGGAGCGCGTCCACCTGTACCGCCCCCCCGGAGCGATCTGGGCGGCATTGCGGTGGCCGTCAC
GGTGATCGCTCTGTGGGCCACCCTGTTCGTCTACGGCCTGTGGTTCGTGAAGCTGCCGTGGGCCCTGA
AGGTGGGCGAGACGGCTACCTCCTGGGCGACGATCGCCGCGGTCTTTTTTTCCCTGGAGTTCCTGTAC
ACCGGCCTGTTTATCACGACCCACGACGCCATGCACGGCACCATTGCGCTCCGCAACCGCCGCCTGAA
CGACTTCCTGGGCAACCTGGCCATCAGCCTGTACGCGTGGTTTGACTACAGCGTGCTGCACCGCAAGC
ATTGGGAGCATCACAACCACACGGGCGAGCCGCGCGTGGACCCGGACTTCCACCGCGGCAACCCCAA
CCTGGCGGTGTGGTTCGCCCAGTTTATGGTGAGCTACATGACGCTGAACCAGTTCCTGAAGATTGCGG
TGTGGAGCAACCTGCTGCTGCTGGCCGGCGCGCCGCTGGCGAACCAGCTGCTGTTTATGACCGCCGC
GCCTATCCTGTCCGCTTTCCGCCTGTTCTACTACGGTACGTACGTCCCTCACCATCCCGAGAAGGGGCA
CACCGGCGCGATGCCGTGGCAGGTGAGCCGCACGTCCTCGGCCAGCCGCCTGCAGAGCTTCCTGACC
TGCTACCACTTCGACCTGCACTGGGAGCACCACCGGTGGCCTTACGCGCCGTGGTGGGAGCTGCCGA
AGTGCCGCCAGATCGCCCGCGGCGCGGCCCTGGCGGTCGACAAGCTTATGGTGAGCAAGGGCGAGGA
GCTGTTCACCGGGGTGGTGCCCATCCTGGTCGAGCTGGACGGCGACGTAAACGGCCACAAGTTCAGC
GTGTCCGGCGAGGGCGAGGGCGATGCCACCTACGGCAAGCTGACCCTGAAGTTCATCTGCACCACCG
GCAAGCTGCCCGTGCCCTGGCCCACCCTCGTGACCACCTTCACCTACGGCGTGCAGTGCTTCAGCCGC
TACCCCGACCACATGAAGCAGCACGACTTCTTCAAGTCCGCCATGCCCGAAGGCTACGTCCAGGAGC
GCACCATCTTCTTCAAGGACGACGGCAACTACAAGACCCGCGCCGAGGTGAAGTTCGAGGGCGACAC
CCTGGTGAACCGCATCGAGCTGAAGGGCATCGACTTCAAGGAGGACGGCAACATCCTGGGGCACAAG
CTGGAGTACAACTACAACAGCCACAACGTCTATATCATGGCCGACAAGCAGAAGAACGGCATCAAGG
TGAACTTCAAGATCCGCCACAACATCGAGGACGGCAGCGTGCAGCTCGCCGACCACTACCAGCAGAA
CACCCCCATCGGCGACGGCCCCGTGCTGCTGCCCGACAACCACTACCTGAGCACCCAGTCCGCCCTG
AGCAAAGACCCCAACGAGAAGCGCGATCACATGGTCCTGCTGGAGTTCGTGACCGCCGCCGGGATCA
CTCACGGCATGGACGAGCTGTACAAGTAAAGCGGCCCGAATTTCCCCGATCGTTCAAACATTTGGCAA
TAAAGTTTCTTAAGATTGAATCCTGTTGCCGGTCTTGCGATGATTATCATATAATTTCTGTTGAATTACGT
TAAGCATGTAATAATTAACATGTAATGCATGACGTTATTTATGAGATGGGTTTTTATGATTAGAGTCCCGC
AATTATACATTTAATACGCGATAGAAAACAAAATATAGCGCGCAAACTAGGATAAATTATCGCGCGCGG
TGTCATCTATGTTACTAGATCGGGAATTCACTGGCCGTCGTTTTACAACGTCGTGACTGGGAAAACCCT
GGCGTTACCCAACTTAATCGCCTTGCAGCACATCCCCCTTTCGCCAGCTGGCGTAATAGCGAAGAGGC
CCGCACCGATCGCCCTTCCCAACAGTTGCGCAGCCTGAATGGCGAATGGCGCCTGATGCGGTATTTTC
TCCTTACGCATCTGTGCGGTATTTCACACCGCATATGAGCTTGCATGCCGGGCGCGCCAGAAGGAGCG
CAGCCAAACCAGGATGATGTTTGATGGGGTATTTGAGCACTTGCAACCCTTATCCGGAAGCCCCCTGG
CCCACAAAGGCTAGGCGCCAATGCAAGCAGTTCGCATGCAGCCCCTGGAGCGGTGCCCTCCTGATAA
ACCGGCCAGGGGGCCTATGTTCTTTACTTTTTTACAAGAGAAGTCACTCAACATCTTAAAATGGCCAG
GTGAGTCGACGAGCAAGCCCGGCGGATCAGGCAGCGTGCTTGCAGATTTGACTTGCAACGCCCGCAT
TGTGTCGACGAAGGCTTTTGGCTCCTCTGTCGCTGTCTCAAGCAGCATCTAACCCTGCGTCGCCGTTTC
CATTTGCAGGATGCTCGAGATTCGAAGCATGGACGATGCGTTGCGTGCACTGCGGGGTCGGTATCCCG
GTTGTGAGTGGGTTGTTGTGGAGGATGGGGCCTCGGGGGCTGGTGTTTATCGGCTTCGGGGTGGTGGG
CGGGAGTTGTTTGTCAAGGTGGCAGCTCTGGGGGCCGGGGTGGGCTTGTTGGGTGAGGCTGAGCGGC
TGGTGTGGTTGGCGGAGGTGGGGATTCCCGTACCTCGTGTTGTGGAGGGTGGTGGGGACGAGAGGGT

CGCCTGGTTGGTCACCGAAGCGGTTCCGGGGCGTCCGGCCAGTGCGCGGTGGCCGCGGGAGCAGCG
GCTGGACGTGGCGGTGGCGCTCGCGGGGCTCGCTCGTTCGCTGCACGCGCTGGACTGGGAGCGGTGT
CCGTTCGATCGCAGTCTCGCGGTGACGGTGCCGCAGGCGGCCCGTGCTGTCGCTGAAGGGAGCGTCG
ACTTGGAGGATCTGGACGAGGAGCGGAAGGGGTGGTCGGGGGAGCGGCTTCTCGCCGAGCTGGAGC
GGACTCGGCCTGCGGACGAGGATCTGGCGGTTTGCCACGGTGACCTGTGCCCGGACAACGTGCTGCT
CGACCCTCGTACCTGCGAGGTGACCGGGCTGATCGACGTGGGGCGGGTCGGCCGTGCGGACCGGCAC
TCCGATCTCGCGCTGGTGCTGCGCGAGCTGGCCCACGAGGAGGACCCGTGGTTCGGGCCGGAGTGTT
CCGCGGCGTTCCTGCGGGAGTACGGGCGCGGGTGGGATGGGGCGGTATCGGAGGAAAAGCTGGCGTT
TTACCGGCTGTTGGACGAGTTCTTCTGACATATGGTGCACTCTCAGTACAATCTGCTCTGATGCCGCAT
AGTTAAGCCAGCCCCGACACCCGCCAACACCCGCTGACGCGCCCTGACGGGCTTGTCTGCTCCCGGC
ATCCGCTTACAGACAAGCTGTGACCGTCTCCGGGAGCTGCATGTGTCAGAGGTTTTCACCGTCATCAC
CGAAACGCGCGAGACGAAAGGGCCTCGTGATACGCCTATTTTTATAGGTTAATGTCATGATAATAATGG
TTTCTTAGACGTCAGGTGGCACTTTTCGGGGAAATGTGCGCGGAACCCCTATTTGTTTATTTTTCTAAAT
ACATTCAAATATGTATCCGCTCATGAGACAATAACCCTGATAAATGCTTCAATAATATTGAAAAAGGAA
GAGTATGAGTATTCAACATTTCCGTGTCGCCCTTATTCCCTTTTTTGCGGCATTTTGCCTTCCTGTTTTTG
CTCACCCAGAAACGCTGGTGAAAGTAAAAGATGCTGAAGATCAGTTGGGTGCACGAGTGGGTTACAT
CGAACTGGATCTCAACAGCGGTAAGATCCTTGAGAGTTTTCGCCCCGAAGAACGTTTTCCAATGATGA
GCACTTTTAAAGTTCTGCTATGTGGCGCGGTATTATCCCGTATTGACGCCGGGCAAGAGCAACTCGGTC
GCCGCATACACTATTCTCAGAATGACTTGGTTGAGTACTCACCAGTCACAGAAAAGCATCTTACGGATG
GCATGACAGTAAGAGAATTATGCAGTGCTGCCATAACCATGAGTGATAACACTGCGGCCAACTTACTTC
TGACAACGATCGGAGGACCGAAGGAGCTAACCGCTTTTTTGCACAACATGGGGGATCATGTAACTCGC
CTTGATCGTTGGGAACCGGAGCTGAATGAAGCCATACCAAACGACGAGCGTGACACCACGATGCCTG
TAGCAATGGCAACAACGTTGCGCAAACTATTAACTGGCGAACTACTTACTCTAGCTTCCCGGCAACAA
TTAATAGACTGGATGGAGGCGGATAAAGTTGCAGGACCACTTCTGCGCTCGGCCCTTCCGGCTGGCTG
GTTTATTGCTGATAAATCTGGAGCCGGTGAGCGTGGGTCTCGCGGTATCATTGCAGCACTGGGGCCAG
ATGGTAAGCCCTCCCGTATCGTAGTTATCTACACGACGGGGAGTCAGGCAACTATGGATGAACGAAATA
GACAGATCGCTGAGATAGGTGCCTCACTGATTAAGCATTGGTAACTGTCAGACCAAGTTTACTCATATA
TACTTTAGATTGATTTAAAACTTCATTTTTAATTTAAAAGGATCTAGGTGAAGATCCTTTTTGATAATCTC
ATGACCAAAATCCCTTAACGTGAGTTTTCGTTCCACTGAGCGTCAGACCCCGTAGAAAAGATCAAAGG
ATCTTCTTGAGATCCTTTTTTTCTGCGCGTAATCTGCTGCTTGCAAACAAAAAAACCACCGCTACCAGC
GGTGGTTTGTTTGCCGGATCAAGAGCTACCAACTCTTTTTCCGAAGGTAACTGGCTTCAGCAGAGCGC
AGATACCAAATACTGTCCTTCTAGTGTAGCCGTAGTTAGGCCACCACTTCAAGAACTCTGTAGCACCGC
CTACATACCTCGCTCTGCTAATCCTGTTACCAGTGGCTGCTGCCAGTGGCGATAAGTCGTGTCTTACCG
GGTTGGACTCAAGACGATAGTTACCGGATAAGGCGCAGCGGTCGGGCTGAACGGGGGGTTCGTGCAC
ACAGCCCAGCTTGGAGCGAACGACCTACACCGAACTGAGATACCTACAGCGTGAGCTATGAGAAAGC
GCCACGCTTCCCGAAGGGAGAAAGGCGGACAGGTATCCGGTAAGCGGCAGGGTCGGAACAGGAGAG
CGCACGAGGGAGCTTCCAGGGGGAAACGCCTGGTATCTTTATAGTCCTGTCGGGTTTCGCCACCTCTG
ACTTGAGCGTCGATTTTTGTGATGCTCGTCAGGGGGGCGGAGCCTATGGAAAAACGCCAGCAACGCG
GCCTTTTTACGGTTCCTGGCCTTTTGCTGGCCTTTTGCTCACATGTTCTTTCCTGCGTTATCCCCTGATTC
TGTGGATAACCGTATTACCGCCTTTGAGTGAGCTGATACCGCTCGCCGCAGCCGAACGACCGAGCGCA
GCGAGTCAGTGAGCGAGGAAGCGGAAGAGCGCCCAATACGCAAACCGCCTCTCCCCGCGCGTTGGC
CGATTCATTAATGCAGCTGGCACGACAGGTTTCCCGACTGGAAAGCGGGCAGTGAGCGCAACGCAAT
TAATGTGAGTTAGCTCACTCATTAGGCACCCCAGGCTTTACACTTTATGCTTCCGGCTCGTATGTTGTGT
GGAATTGTGAGCGGATAACAATTTCACACAGGAAACAGCTATGACCATGATTACGCC

Supplementary material S2. Figures of EGFP fluorescence signal of colonies by Inverted Fluorescence Microscope.

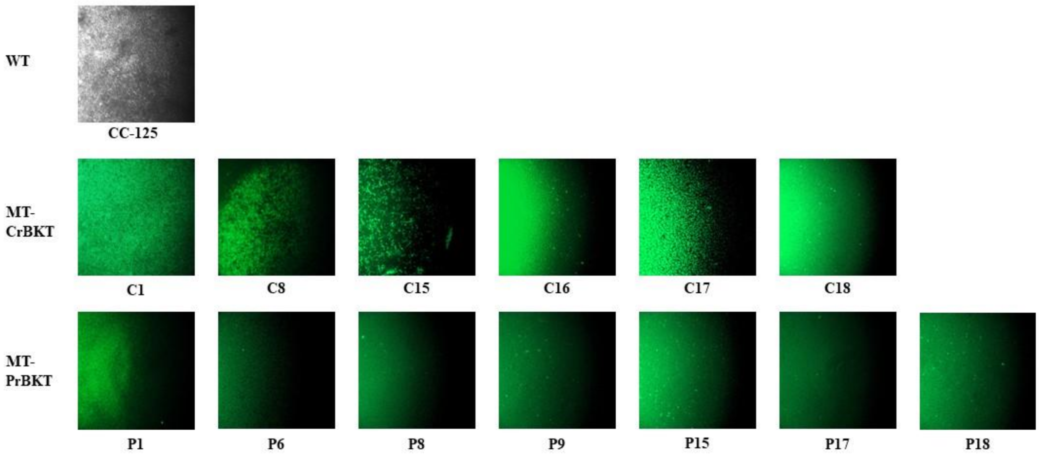

Supplementary material S3. The standard curve of astaxanthin (a) and β-carotenoid (b) and figure of absorption peak for standards and cultures (c-g, Figures of peak area were displayed for one of the replicates of Day 4 for each of the three groups due to too large numbers).

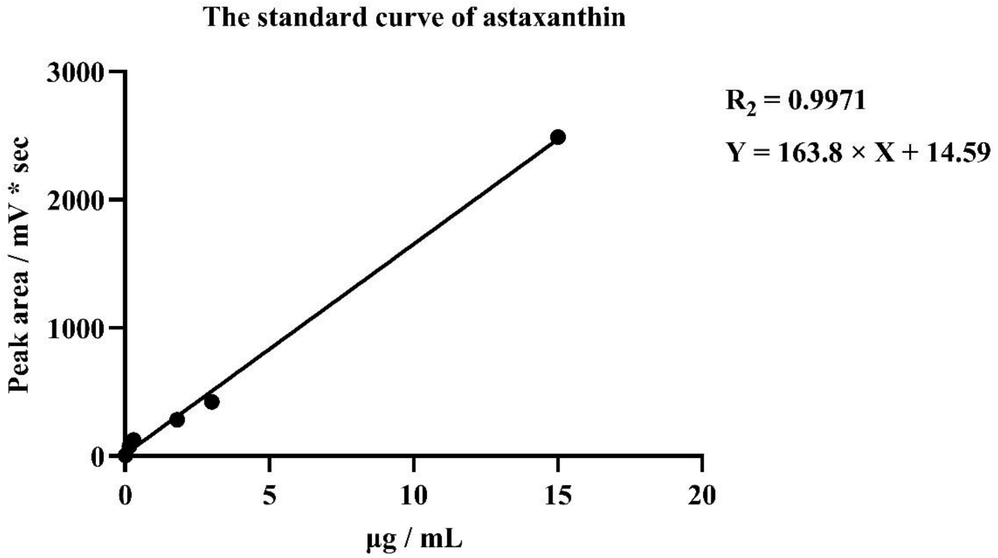

(a) The standard curve of astaxanthin

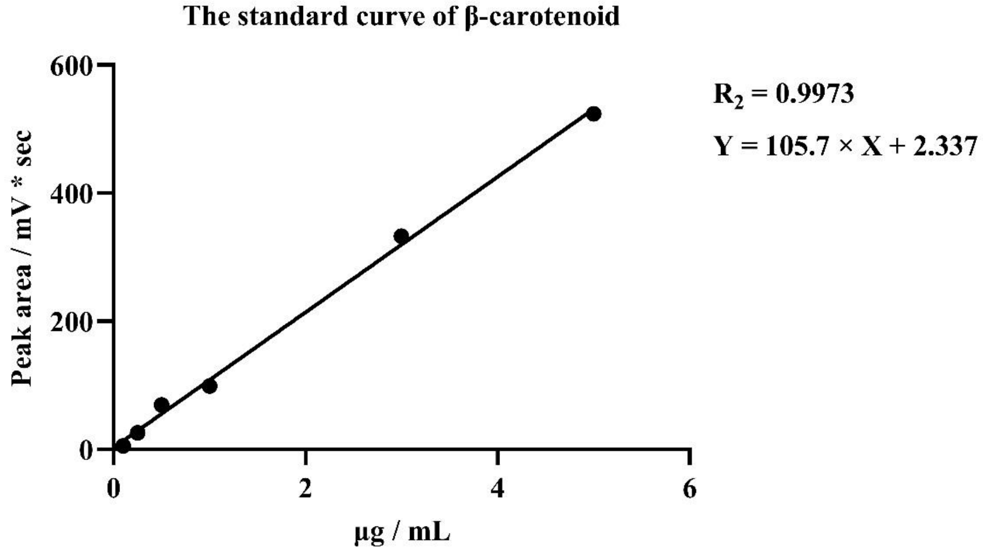

(b) The standard curve of β-carotenoid

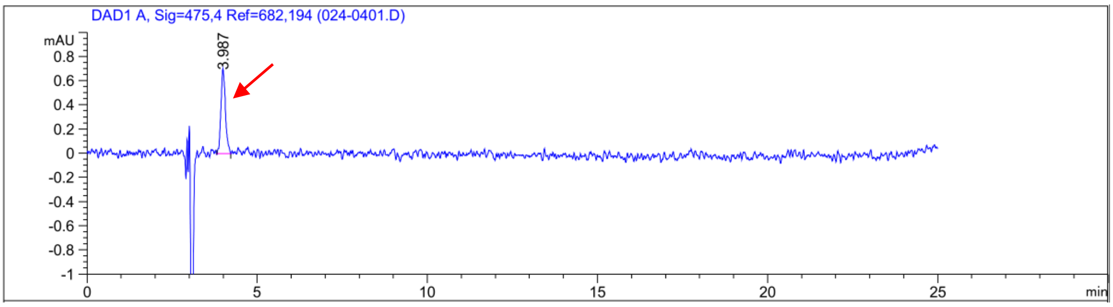

(c) Figure of astaxanthin standards (0.1 μg / mL) peak area

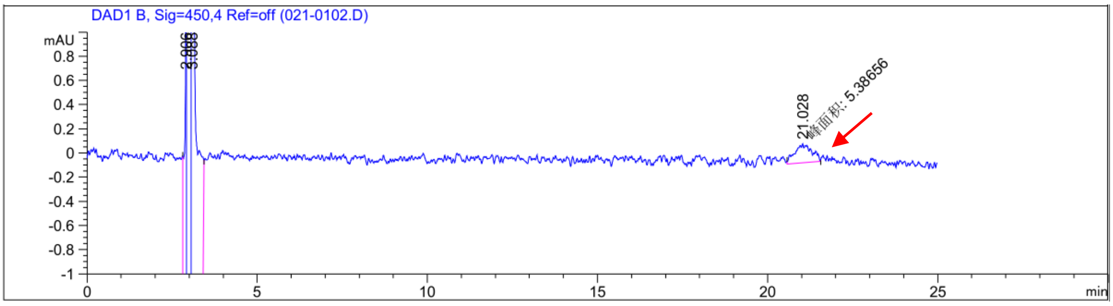

(d) Figure of β-carotenoid standards (0.1 μg / mL) peak area

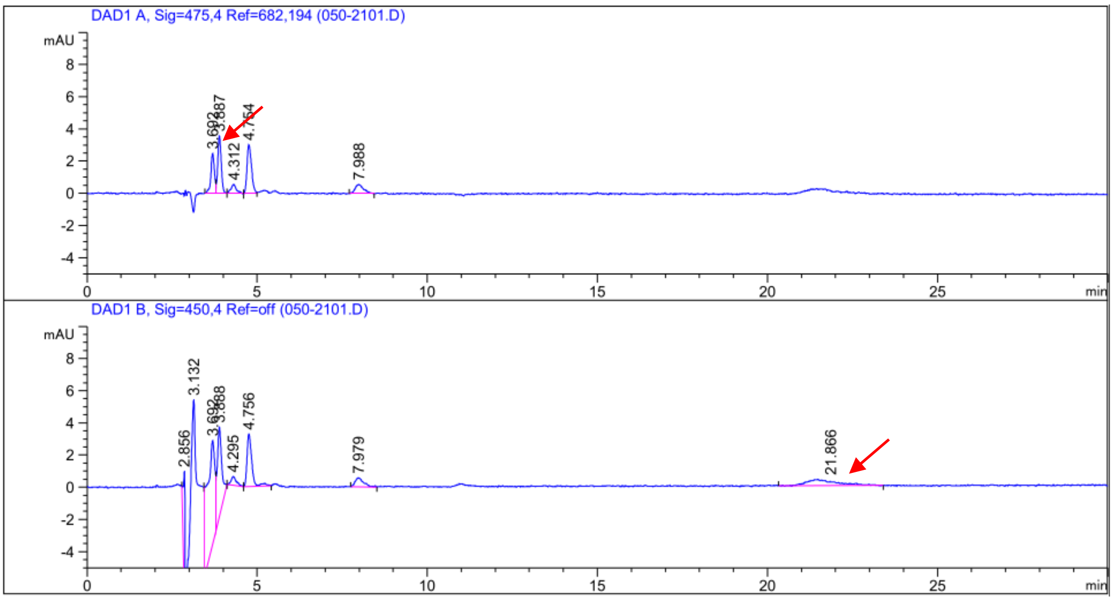

(e) Figure of CC-125 cultures (Day 4) peak area

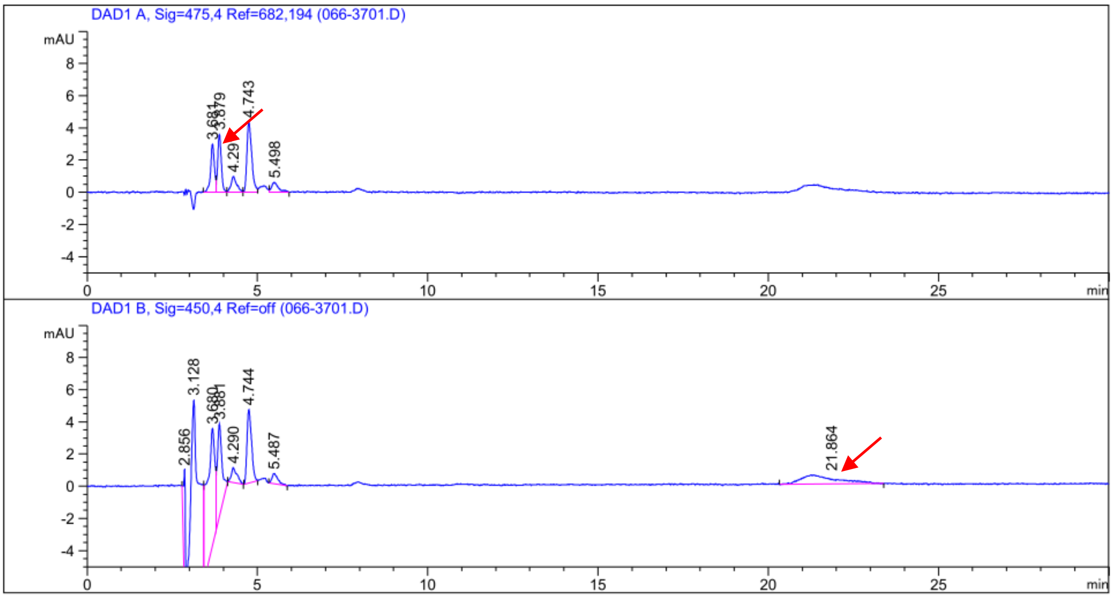

(f) Figure of C18 cultures (Day 4) peak area:

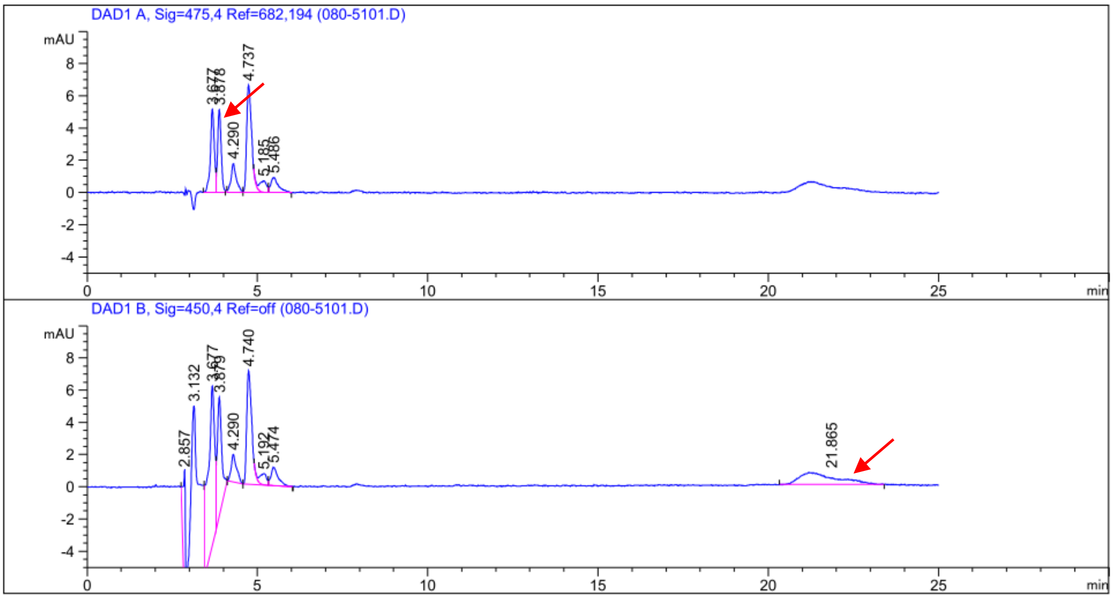

(g) Figure of P1 cultures (Day 4) peak area:

Supplementary material S4. Primer list

Gene Name	ID	Primers		USE
PARA	/	F	TCAACTTGTTGGCGAGATT	The para sequence was amplified from E.coli maintained in our laboratory
		R	GTGGAATTGTGAGCGGATA	
CrBKT in PBI221-CrBKT	/	F	CATGGCGGAGGACATCTTAAA	The CrBKT sequence was amplified in PBI221-CrBKT
		R	ACGTAGGTGCCGTAGTAGAA	
PrBKT in PBI2221-PrBKT	/	F	TCAAGCAGTTCACGAAGCA	The PrBKT sequence was amplified in PBI221-CrBKT
		R	CGTAGTAGAACAGGCGGAAAG	
CrBKT in PBI221-CrBKT	/	F	CCTGTATGCGTGGTTTGATTAC	For qRT-PCR in CrBKT overexpressional colonies
		R	GTCATGTAGGACACCATGAACT	
PrBKT in PBI2221-PrBKT	/	F	GCTGAACCAGTTCCTGAAGAT	For qRT-PCR in PrBKT overexpressional colonies
		R	CGTAGTAGAACAGGCGGAAAG	
BKT	CHLRE_04g215000v5	F	GGCGACGCTGTTTGTCTA	For qRT-PCR in WT, C18, and P1
		R	ATGAAGAGCCCGGTGTAAAG	
CHYB	CHLRE_04g215050v5	F	GGCATCGCCTACATGTTCTT	For qRT-PCR in WT, C18, and P1
		R	TTGTTGGTGTGGTGGATCTG	
LCYE	CHLRE_06g267600v5	F	GGCTTGCCAGGTTGTATCT	For qRT-PCR in WT, C18, and P1
		R	CCCTCGCGGATTTCTTGAT	
ZDS	CHLRE_07g314150v5	F	GTGTTCTTCGGCTGCTACTT	For qRT-PCR in WT, C18, and P1
		R	TGTGTGTGTGCTCCTTGAC	
CHYE	CHLRE_02g142266v5	F	AGTGTTTGTGCCGCTGTA	For qRT-PCR in WT, C18, and P1
		R	AGAGATGATGACGAAGCTCTTG	
ZEP	CHLRE_02g082550v5	F	CGAGGTGTTCAAGGTGAAGATG	For qRT-PCR in WT, C18, and P1
		R	GGCATGACGTAGTCGTTGTT	
