# Supplementary material for: Enhanced Carotenoid Production in Chlamydomonas reinhardtii by Overexpression of Endogenousand Exogenous Beta-Carotene Ketolase (BKT) Genes
Source: Int J Mol Sci. 2023 Jul 13;24(14):11382. doi: 10.3390/ijms241411382 (PMC10379168; doi:10.3390/ijms241411382)
Supplement: Supplementary file 1 [file ijms-24-11382-s001.zip › Supplementary material S5.pdf]

Method

Sample collection and preparation

RNA quantification and qualification

RNA integrity was assessed using the RNA Nano 6000 Assay Kit of the Bioanalyzer 2100 system (Agilent Technologies, CA, USA).

Library preparation for Transcriptome sequencing

Total RNA was used as input material for the RNA sample preparations. Briefly, mRNA was purified from total RNA using poly-T oligo-attached magnetic beads. Fragmentation was carried out using divalent cations under elevated temperature in First Strand Synthesis Reaction Buffer(5X). First strand cDNA was synthesized using random hexamer primer and M-MuLV Reverse Transcriptase(RNase H-). Second strand cDNA synthesis was subsequently performed using DNA Polymerase I and RNase H. Remaining overhangs were converted into blunt ends via exonuclease/polymerase activities. After adenylation of 3' ends of DNA fragments, Adaptor with hairpin loop structure were ligated to prepare for hybridization. In order to select cDNA fragments of preferentially 370~420 bp in length, the library fragments were purified with AMPure XP system (Beckman Coulter, Beverly, USA). Then PCR was performed with Phusion High-Fidelity DNA polymerase, Universal PCR primers and Index (X) Primer. At last, PCR products were purified (AMPure XP system) and library quality was assessed on the Agilent Bioanalyzer 2100 system.

Clustering and sequencing (Novogene Experimental Department)

The clustering of the index-coded samples was performed on a cBot Cluster Generation System using TruSeq PE Cluster Kit v3-cBot-HS (Illumia) according to the manufacturer's instructions. After cluster generation, the library preparations were sequenced on an Illumina Novaseq platform and 150 bp paired-end reads were generated.

Data Analysis

Quality control

Raw data (raw reads) of fastq format were firstly processed through fastp software. In this step, clean data (clean reads) were obtained by removing reads containing adapter, reads containing

ploy-N and low quality reads from raw data. At the same time, Q20, Q30 and GC content the clean data were calculated. All the downstream analyses were based on the clean data with high quality.

Reads mapping to the reference genome

Reference genome and gene model annotation files were downloaded from genome website directly. Index of the reference genome was built using Hisat2 v2.0.5 and paired-end clean reads were aligned to the reference genome using Hisat2 v2.0.5. We selected Hisat2 as the mapping tool for that Hisat2 can generate a database of splice junctions based on the gene model annotation file and thus a better mapping result than other non-splice mapping tools.

Novel transcripts prediction

The mapped reads of each sample were assembled by StringTie (v1.3.3b) (Mihaela Pertea.et al. 2015) in a reference-based approach. StringTie uses a novel network flow algorithm as well as an optional de novo assembly step to assemble and quantitate fulllength transcripts representing multiple splice variants for each gene locus.

Quantification of gene expression level

featureCounts v1.5.0-p3 was used to count the reads numbers mapped to each gene. And then FPKM of each gene was calculated based on the length of the gene and reads count mapped to this gene. FPKM, expected number of Fragments Per Kilobase of transcript sequence per Millions base pairs sequenced, considers the effect of sequencing depth and gene length for the reads count at the same time, and is currently the most commonly used method for estimating gene expression levels.

Differential expression analysis

(For DESeq2 with biological replicates) Differential expression analysis of two conditions/groups (two biological replicates per condition) was performed using the DESeq2 R package (1.20.0). DESeq2 provide statistical routines for determining differential expression in digital gene expression data using a model based on the negative binomial distribution. The resulting P-values were adjusted using the Benjamini and Hochberg's approach for controlling the false discovery rate . Genes with an adjusted P-value <=0.05 found by DESeq2 were assigned as differentially expressed.

(For edgeR without biological replicates) Prior to differential gene expression analysis, for each sequenced library, the read counts were adjusted by edgeR program package through one scaling normalized factor. Differential expression analysis of two conditions was performed using the edgeR R package (3.22.5). The P values were adjusted using the Benjamini & Hochberg method. Corrected P-value of 0.05 and absolute foldchange of 2 were set as the threshold for significantly differential expression.

GO and KEGG enrichment analysis of differentially expressed genes

Gene Ontology (GO) enrichment analysis of differentially expressed genes was implemented by the clusterProfiler R package, in which gene length bias wascorrected. GO terms with corrected Pvalue less than 0.05 were considered significantly enriched by differential expressed genes. KEGG is a database resource for understanding high-level functions and utilities of the biological system, such as the cell, the organism and the ecosystem, from molecular-level information, especially large-scale molecular datasets generated by genome sequencing and other high-through put experimental technologies (http://www.genome.jp/kegg/). We used clusterProfiler R package to test the statistical enrichment of differential expression genes in KEGG pathways.

Gene Set Enrichment Analysis

Gene Set Enrichment Analysis (GSEA) is a computational approach to determine if a pre-defined Gene Set can show a significant consistent difference between two biological states. The genes were ranked according to the degree of differential expression in the two samples, and then the predefined Gene Set were tested to see if they were enriched at the top or bottom of the list. Gene set enrichment analysis can include subtle expression changes. We use the local version of the GSEA analysis tool http://www.broadinstitute.org/gsea/index.jsp, GO, KEGG data set were used for GSEA independently.

SNP analysis

GATK (v4.1.1.0) software was used to perform SNP calling. Raw vcf files were filtered with GATK standard filter method and other parameters (cluster:3; WindowSize:35; QD < 2.0 ; FS > 30.0; DP < 10.

AS analysis

Alternative Splicing is an important mechanism for regulate the expression of genes and the variable of protein. rMATS(4.1.0) software was used to analysis the AS event.

PPI analysis of differentially expressed genes

PPI analysis of differentially expressed genes was based on the STRING database, which known and predicted Protein-Protein Interactions.

Weighted correlation network analysis

WGCNA (Weighted correlation network analysis) is a systematic biological method used to describe the gene association modes among different samples. it can be used to identify gene sets that are highly synergistic changed, and identify candidate biomarkers or therapeutic targets based on the coherence of gene sets and the correlation between gene sets and phenotypes. The R package WGCNA is a set of functions used to calculate various weighted association analysis, which can be used for network construction, gene screening, gene cluster identification, topological feature calculation, data simulation and visualization. WGCNA is suitable for multisample data. Generally, more than 15 samples are required. One input file is sample information, that is, a matrix describing the traits of the sample: the traits used for association analysis must be numeric; If it is a regional or categorical variable, it needs to be converted to a 0-1 matrix. The other is gene expression data. For transcriptome sequencing, FPKM can be used as gene expression data.

No.	Sample Name	RNA concentration (ng/ul)	Volume (ul)	Total RNA (ug)	RNA Integrity Value
1	WT_1	44	35	1.54	6.6
2	WT_2	28	35	0.98	6.9
3	WT_3	38	35	1.33	6.4
4	C18_1	18	35	0.63	4
5	C18_2	29	35	1.015	4.1
6	C18_3	16	35	0.56	6.3
7	P1-1	59	30	1.77	4.7
8	P1-2	82	30	2.46	4.4
9	P1-3	104	35	3.64	4

Footnote：RNA quality for transcriptome.
